# Capping motifs in antimicrobial peptides and their relevance for improved biological activities

**DOI:** 10.3389/fchem.2024.1382954

**Published:** 2024-05-30

**Authors:** José Brango-Vanegas, Michel Lopes Leite, Maria L. R. Macedo, Marlon H. Cardoso, Octávio Luiz Franco

**Affiliations:** ^1^ Centro de Análises Proteômicas e Bioquímicas, Programa de Pós-Graduação em Ciências Genômicas e Biotecnologia, Universidade Católica de Brasília, Brasília, Brazil; ^2^ S-inova Biotech, Programa de Pós-Graduação em Biotecnologia, Universidade Católica Dom Bosco, Campo Grande, Brazil; ^3^ Departamento de Biologia Molecular, Instituto de Ciências Biológicas, Universidade de Brasília, Campus Darcy Ribeiro, Brasília, Brazil; ^4^ Laboratório de Purificação de Proteínas e suas Funções Biológicas, Universidade Federal de Mato Grosso do Sul, Cidade Universitária, Campo Grande, Brazil

**Keywords:** capping motif, antimicrobial peptides, metallo-AMPs, amino acid motifs, secondary structure

## Abstract

N-capping (N-cap) and C-capping (C-cap) in biologically active peptides, including specific amino acids or unconventional group motifs, have been shown to modulate activity against pharmacological targets by interfering with the peptide’s secondary structure, thus generating unusual scaffolds. The insertion of capping motifs in linear peptides has been shown to prevent peptide degradation by reducing its susceptibility to proteolytic cleavage, and the replacement of some functional groups by unusual groups in N- or C-capping regions in linear peptides has led to optimized peptide variants with improved secondary structure and enhanced activity. Furthermore, some essential amino acid residues that, when placed in antimicrobial peptide (AMP) capping regions, are capable of complexing metals such as Cu^2+^, Ni^2+^, and Zn^2+^, give rise to the family known as metallo-AMPs, which are capable of boosting antimicrobial efficacy, as well as other activities. Therefore, this review presents and discusses the different strategies for creating N- and C-cap motifs in AMPs, aiming at fine-tuning this class of antimicrobials.

## 1 Introduction

The affinity of antimicrobial peptides (AMPs) to the microorganism’s membrane have been associated with the net charge and amphiphilicity (Tossi et al., 2000). In most cases, short AMPs sequences with cationic and hydrophobic side chains undergo a coil-to-helix transition from aqueous environments to membrane-like condition (*e.g.*, bilayer phospholipids) (Li et al., 2022). These cationic α-helix AMPs usually as stable or unstable pore formers. The unstable pores can generate a reorganization of the membrane surface and consequently reorient proteins and receptors, resulting in a change in the transmembrane potential, causing an imbalance and cell death (Łoboda et al., 2018; Donaghy et al., 2023). However, just as helices can be formed and incorporated within the membrane to form pores, so can several different types of amphipathic structures be formed, not necessarily imposed by a strict primary or secondary structural organization (Tossi et al., 2000). As well as helical amphipathic, resulting in part from punctual interactions of the residues within a given sequence, other environmental factors such as pH conditions, ionic strength, and the presence of divalent cations are responsible for AMP activity effectiveness (Donaghy et al., 2023).

In addition to their membranolytic activity on microbes, α-helix peptides can cross the plasma membrane, through the spontaneous translocation mechanism. They engage with intracellular targets, including nucleic acids such as DNA and RNA, preventing protein synthesis, and bind to other molecules, such as enzymes and proteases, affecting important cellular functions (Łoboda et al., 2018). Simultaneously, they exhibit minimal side effects in mammalian cells (Alexander et al., 2017; Li et al., 2022). Implementing strategies to ensure that AMPs establish α-helices to improve their interaction with biological targets, particularly microbes, is crucial in the development of active peptides. The stability of the secondary α-helix is maintained by intramolecular hydrogen bonds formed between the amide hydrogen and carbonyl oxygen atoms in the peptide backbone at positions *i* and *i+4* (Kanbayashi et al., 2022). The emergence of helicity stems from the amino acid residues’ primary sequence and their interaction with the surrounding environment (dos Santos Cabrera et al., 2019). The capping motifs, which have a propensity to generate α-helices in a peptide backbone, are well-documented features present in the secondary structures of proteins and peptides (Kier et al., 2010). These strategies have been a starting point and are used to improve AMPs’ biological activities.

Capping motifs are extremely important for the stabilization of the structure and, consequently, for the activity of AMPs (Park et al., 2004). Specifically, peptides that form α-helices, N-cap, and C-cap motifs, formed by a set of amino acids in sequence located in the terminal parts of the secondary structure, influence the stability of the helical structure. This stabilization results from additional and unconventional hydrogen bonds, as they present dihedral angles different from those found inside the helix (De Rosa et al., 2022). In other words, the capping phenomenon occurs through the contributions of hydrogen donor and acceptor groups, accompanied by hydrophobic interactions at the ends of the helix, as well as polar residues in the helix (Aurora and Rose, 1998). The participating amino acids experience a loss of free energy, favoring the folding and stability of the helical structure (Serrano and Fersht, 1989).

For example, the presence of a Lys residue at the *C*-terminus is responsible for stabilizing helix formation in polyalanine peptides with seven or more residues (Zabuga and Rizzo, 2015). N-capping motifs have been shown to promote amphipathic helical peptides’ interaction with hydrophobic surfaces, dramatically altering the hydrophobicity characteristics of individual amino acid residues (Spicer et al., 2014). Recently, an unusual N-capping motif was identified and formed by an asparagine-lysine-proline (Asn-Lys-Pro) motif. This motif is present in the PaDBS1R7 peptide and has a role in the hybrid structural formation (coil/N-cap/α-helix), contributing to a diverse range of biological activities (Cardoso et al., 2022).

Therefore, in the following topics, we will address the importance of the capping motifs for the stabilization of the secondary structures of AMPs, as well as how it can influence the activity of these molecules. Furthermore, we will discuss motifs composed of amino acid sequences and explore unusual modifications on terminal regions that facilitate helical structuring and enhance activity. Additionally, the capping effects due to unusual groups that initiate the nucleation of peptides as helices will be explored. We will also cover coordination interactions between AMPs and metals, resulting in macrostructures that enhance peptide activity. Employing these motifs is a promising approach for the development of more effective AMPs.

## 2 Improving the structure of AMPs to enhance their activity

In spite of recent progress, the translational clinical development of AMPs faces challenges, leading to delays in current design strategies (Jiang et al., 2021). A key strategy involves introducing capping motifs, taking advantage of changes in structural features within peptides to promote nucleation and stabilization of α-helix structures. This approach is crucial because helices play a pivotal role as secondary structures, influencing the activity of AMPs through molecular recognition.

The importance of helical structure allows AMPs to be continuously exploited for potential individual use or in combination with established antibiotics, especially in the new era of treating multidrug resistant bacteria affecting both human and animal health (Lei et al., 2019; Fu et al., 2023). Highlighting their advantages, AMPs offer a slower emergence of resistance, rapid lethal action, and effective control of biofilms, positioning them as optimal candidates for treatment of drug-resistant pathogens (Magana et al., 2020). Furthermore, these peptides have shown immunomodulatory effects, either by diminishing the inflammatory response triggered by endotoxins, inducing the synthesis of pro-inflammatory factors, or eliciting the secretion of cytokines (Lei et al., 2019).

Different chemical modifications, including specific substitutions and/or residue additions to the primary AMP sequence can be used, along with computational approaches to analyze physicochemical and structural properties from the combinatorial library, thus providing analogs with improved activity (Ong et al., 2014; de Oliveira et al., 2023). Among the possible strategies, we can cite the substitution of specific residues, total or specific change of the stereochemistry of amino acids, as well as *N*- and *C*-terminal modification, cyclization, and stapling (de Oliveira et al., 2023). Other classic ways to improve the activity and performance of AMPs are the insertion of unusual amino acids, tricyclic groups, and modifications on the scaffold to generate peptidomimetics (Petri et al., 2022). Hybrid computational methods, such as the combination of different experimental data with molecular dynamics simulations (Mondal et al., 2023), can be employed to predict information to improve secondary structures in peptides. Additionally, artificial intelligence algorithms, such as machine and deep learning (Jiang et al., 2023; Yue et al., 2024), as well as geometric deep learning (Fernandes et al., 2023), contribute to this advancement. This approach has recently become a valuable tool to understand the structure-activity relationship of AMPs and to design the next-generation of peptides with improved properties.

With these strategies, it is possible to solve problems such as susceptibility to proteases, but also in specific cases, three-dimensional structuring can be favored, such as α-helix, seeking to improve molecular interactions of AMPs with biological targets such as proteins, receptors, nucleic acids, and other biomolecules of interest related to infectious diseases caused by microbes.

## 3 Capping motif effects due to unusual groups for AMP optimization

The insertion of specific amino acid motifs into N-cap or C-cap regions has been observed to induce the formation of AMP helices, consequently enhancing their activity against molecular targets. This phenomenon is attributed to the intramolecular interactions among the residues within the peptide backbone (Aurora and Rose, 1998), which provide the necessary balance of rigidity and flexibility to facilitate the structural conformation of a helix (Babii et al., 2017). Natural amino acids play a significant role in enhancing the performance of AMPs, as the side chains within peptides and proteins contribute to molecular recognition through steric and electronic effects (King et al., 2021), but other modifications involving unusual groups can also contribute to their performance.

The capping motif phenomenon in linear peptides also encompasses terminal modifications (Table 1). These modifications can prevent chain degradation by reducing the vulnerability of peptide bonds to proteolytic cleavage (Behrouz et al., 2023; Ding et al., 2023). Furthermore, specific modifications at the ends of sequences employ unconventional conformational motifs, forming intramolecular H-bond pairs to enhance both the structural characteristics and activity of AMPs (Aurora and Rose, 1998).

**TABLE 1:** Structure, capping type and interaction of some unusual groups described in the literature for AMP optimization.

Template/group/name	Structures	Capping type	Interactions Cap/Function	Helix type	References
*N*-monosubstitutions into *C*-terminal amidations	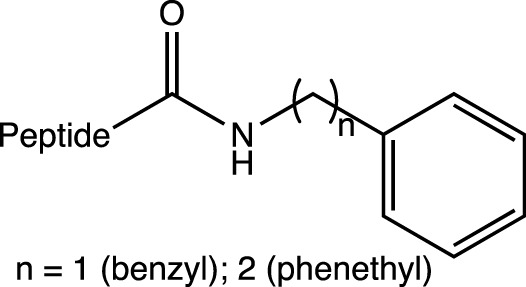	C-cap	Resistance to proteases	-	Svenson et al. (2008)
*N,N*-disubstitutions into *C*-terminal amidations	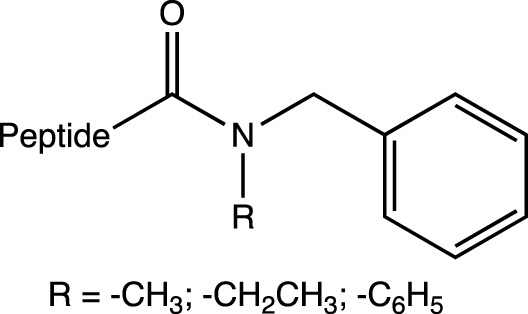	C-cap	Resistance to proteases	-	Svenson et al. (2008)
Chem	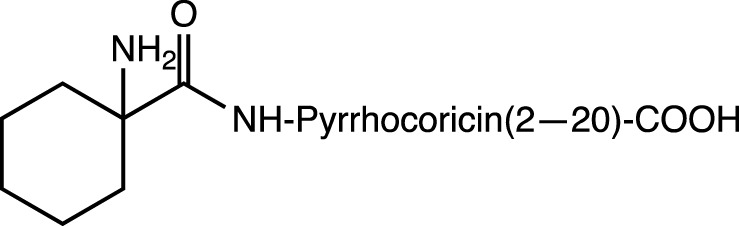	N-cap	Resistance to proteases, as well as induction of helix formation by chair conformation due to the cyclohexane structure	hybrid 3_10_-α helices	Pasupuleti et al. (2009)
Dap (Ac)	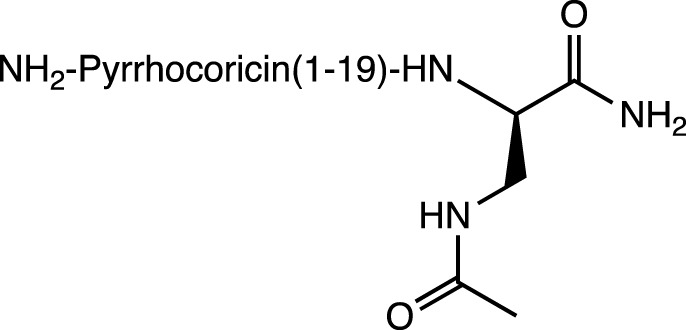	C-cap	Resistance to proteases	-	Pasupuleti et al. (2009)
Two prolines linked by a thiomethylene unit (-CH_2_-S-)	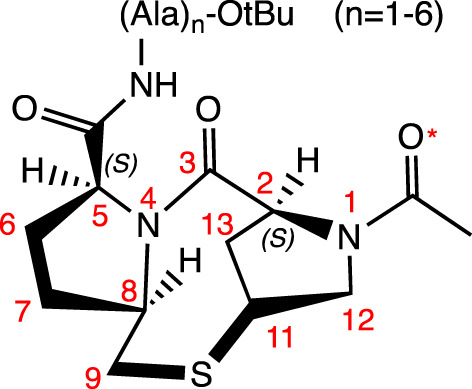	N-cap	H-Bond between NH(Ala-1) and C=O* (acetamide) (*i, i+3*); C=O in the C-3 and NH(Ala-2) (*i, i+2*) and NH(Ala-3) (*i, i+3*). Observed by NOE and ROESY experiments	hybrid 3_10_-α helices	Kemp et al. (1991)
Three prolines linked by two thiomethylene units (-CH_2_-S-)	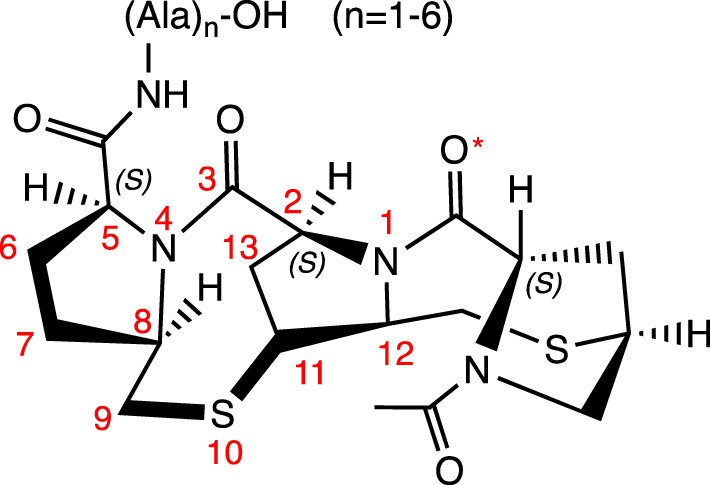	N-cap	H-Bonds between: NH(Ala-1) and C=O* (Pro) (*i, i+3*); Oxygen in the C-3 and NH(Ala-2) (*i, i+2*) and NH(Ala-3) (*i, i+3*). Observed by NOE and ROESY experiments	hybrid 3_10_-α helices	Kemp and Rothman (1995)
ProM-5	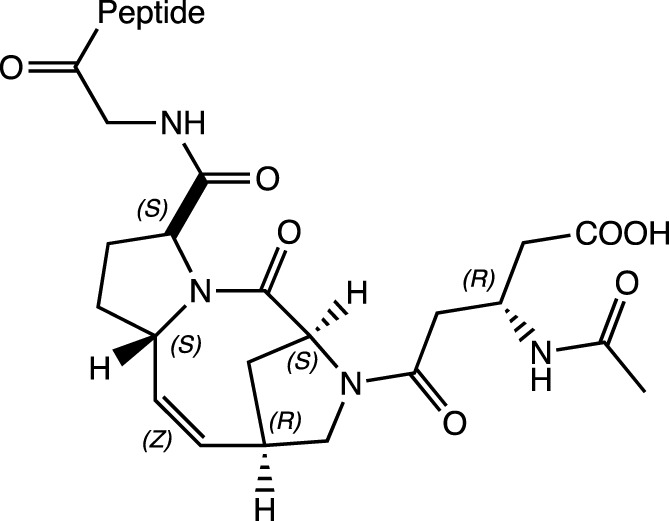	N-cap	H-Bonds between: NH (Gly) and carboxylate; C=O of *N*-acetyl-β-homo-Asp and NH(amino acid-1) (*i, i+4*); C=O in the first proline and NH(amino acid-2) (*i, i+3*). Observed by CD and 2D NMR experiments	α-helix	Hack et al. (2013)
S,S-9-O	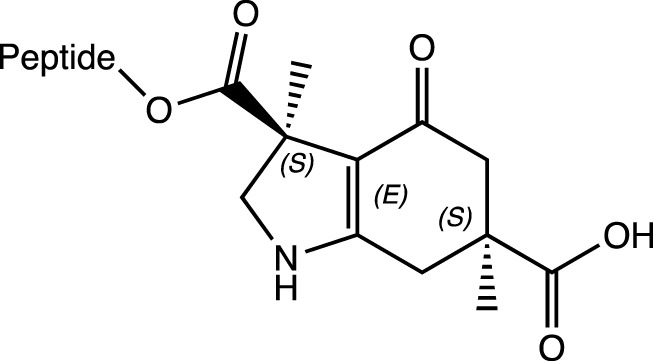	N-cap	Induction to α-helical conformation (between 50% and 75% helicity). Observed by CD	α-helix	Austin et al. (1997)
Complex bicyclic	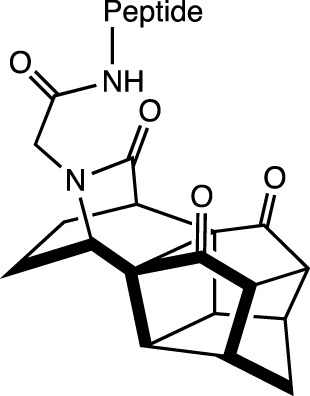	N-cap	H-Bond between NH (first amino acid) and the last of the carbonyls	α-helix	Mahon and Arora (2012)

Dap (Ac): N-acetyl-2, 3-diamino propionic acid; Chem: 1-amino-cyclohexane-carboxylic acid.

Among the most commonly employed modifications are *N*-terminal acetylation and *C*-terminal amidation strategies, aimed at enhancing the conformational stability and availability of active AMPs, while maintaining or enhancing their antimicrobial potency (Mura et al., 2016; Mwangi et al., 2023). These are crucial, especially considering the susceptibility of AMPs to degradation by exopeptidases (Magana et al., 2020). For instance, the *N*-terminal acetylation can facilitate helix nucleation through interactions with the side chain or backbone hydrogen donor of Arg in Ac-Leu-Leu-Arg motifs (Chang et al., 2000); as well as the *N*-terminal amidation in modelin-5 (Lys-Leu-Ala-Lys-Lys-Leu-Ala-Lys-Leu-Ala-Lys-Leu-Ala-Lys-Ala-Leu), contributes to stabilizing helix formation, leading to enhanced levels of amphiphilic helix at a lipid interface, and increasing the efficacy approximately 10-fold in tests against *Enterococcus coli* when compared to peptide not amidated (Dennison and Phoenix, 2011).

In short peptides (of the type Arg-X-Arg-Y, where X is the 4-phenylphenylalanine residue), stability and resistance against trypsin increase when *N*-monosubstitutions (*N*-benzyl and *N*-phenethyl) and *N,N*-disubstitutions (*N*-methyl-N-benzyl; *N*-ethyl-*N*-benzyl, and *N,N*-dibenzyl) are introduced into *C*-terminal amidations (in Y). It should be noted that monosubstituted derivatives with *N*-phenethylamide groups presented good stabilities in various peptides that have X with different aromatic residues (Svenson et al., 2008).

Pyrrocoricin is an AMP from insects that excels against gram-negative bacteria. The peptide has the sequence Val-Asp-Lys-Gly-Ser-Tyr-Leu-Pro-Arg-Pro-Thr(X)-Pro-Pro-Arg-Pro-Ile-Tyr-Asn-Arg-Asn, where X is the disaccharide radical 2-(acetylamino)-2-deoxy-3-O-β-D-galactopyranosyl-α-*D*-galactopyranosyl anchored to the oxygen of Thr-11. Pyrrocoricin analogues were synthesized by Otvos et al. (2000) using the linear sequence without the disaccharide moiety and making changes to the *N*- and *C*-termini to improve its resistance to proteases. Additional amino acids were added at the *N*-terminus, along with acetylation (Ac-Lys-, Ac-Lys-Val-Asp-Lys-, Ac-Arg-), as well as the addition of the Chem group (1-amino-cyclohexane-carboxylic acid). At the *C*-terminus, the Dap (Ac) group (*N*-acetyl-2,3-diamino propionic acid) was added. The results indicate that modification at either end of the termini resulted in a decrease in the antibacterial efficacy of the parent pyrrocoricin. Among the peptides modified at the *N*- or *C*-termini, those with the Chem group at the *N*-terminal and the Dap (Ac) group at the *C*-terminal appear to retain some of the antibacterial activity of the parental pyrrocoricin. An analogue with both modifications showed high potency against bacteria and a lack of toxicity *in vivo*. The effects of protecting groups at the *N*- and *C*-termini play a crucial role in the stability of the peptide in the presence of proteases (Otvos et al., 2000). Peptides containing unusual *N*-terminal amino acids, such as 1-aminocyclopentane-1-carboxylic acid (Acc5) and 1-aminocyclohexane-1-carboxylic acid (Chem group), necessarily adopt folded structural conformations, in the 3_10_/α-helical regions of the conformational space (Santini et al., 1988; Valle et al., 1988). In the case of the Chem residue, the cyclohexane structure provides a perfect chair conformation, with the amino group in the peptidic bond located axially and the carboxylate group in the equatorial position. This arrangement favors interactions at the beginning of the *N*-terminus and promotes a helical structure in the backbone (Paul et al., 1986; Valle et al., 1988).

To enhance their resistance to high salt concentrations, Chu et al. (2013) incorporated 1 to 3 repeats of β-naphthylalanine (NaI) at the *C*-terminus of the Trp-rich synthetic S1 AMP (Ac-Lys-Lys-Trp-Arg-Lys-Trp-Leu-Ala-Lys-Lys-NH_2_). They observed that all three peptides (S1-NaI, S1-NaI-NaI, and S1-NaI-NaI-NaI) were more potent than the unmodified peptide, and the ones with two and three NaI residues still retained their antibacterial activities even with the addition of 300 mM NaCl. Moreover, the peptide with three NaI residues maintained almost 100% integrity after 8 h in bovine calf serum (Chu et al., 2013). Previously, the insertion of motifs containing 2 and 5 Trp into the *C*-terminus of kininogen-derived AMPs, including Lys-Asn-Lys-Gly-Lys-Lys-Asn-Gly-Lys-His (KNK10), Gly-Lys-His-Lys-Asn-Lys-Gly-Lys-Lys-Asn-Gly-Lys-His-Asn-Gly-Trp-Lys (GKH17), and His-Lys-His-Gly-His-Gly-His-Gly-Lys-His-Lys-Asn-Lys-Gly-Lys-Lys-Asn (HKH17), resulted in an enhanced antimicrobial effect against microbes (*S. aureus*, *E. coli*, and *C. albicans*). Additionally, the modified peptides exhibited robust stability against proteolytic degradation by staphylococcal aureolysin V8 proteinase and human leukocyte elastase (Pasupuleti et al., 2009; Schmidtchen et al., 2009). Hydrophobic residues, such as Trp, Phe or β-naphthylalanine, are compelling choices for the terminal positions in AMPs due to their bulky, aromatic, and polarizable nature. These residues interact with the phospholipid membrane, serving as anchors for the peptide. When incorporated into highly cationic AMP sequences, this anchoring effect promotes the formation of membrane defects and facilitates rupture (Pasupuleti et al., 2009; Chu et al., 2013).

Although some unusual groups located at the peptide’s termini can prevent this degradation, their insertion can also favor helicity, as is the case of cyclic proline mimetic motifs, which can favor the nucleation of the α-helix when inserted in the *N-*terminal region. As examples, there are templates with two and three prolines, each linked to the other by a thiomethylene unit (-CH_2_-S-) in the *N*-terminus of polyalanine peptides. Both bicyclic templates serve as powerful motifs for initiating helix formation. These motifs form an H-bond (*i, i+3*) between the NH(Ala-1) group and the carbonyl group of acetamide (for the template with two prolines) and the Pro (in the template with three prolines); as well as two additional H-bonds formed between the C=O group of the first Pro and the NH groups of Ala-2 (*i, i+2*) and Ala-3 (*i, i+3*) (Kemp et al., 1991; Kemp and Rothman, 1995).

A synthetic tricyclic motif, denominated ProM-5 was synthesized through the stereoselective introduction of a vinylidene bridge into a diproline unit. ProM-5 acts as a powerful structure for the nucleation of α-helix formation in a linear polypeptide chain when incorporated as an N-cap. The vinylidene bridge restricts the flexibility of the 8-membered ring to adopt a favorable conformation by the addition of an *N*-acetyl-β-homo-Asp residue located in the *N*-terminus in the last proline. This conformation allows and induces the formation of intermolecular interactions throughout the peptide chain (H-bonds: NH (Gly) and carboxylate; C=O of *N*-acetyl-β-homo-Asp and NH (amino acid-1) (*i, i+4*); C=O in the first proline and NH (amino acid-2) (*i, i+3*)) (Hack et al., 2013).

Another case involves using semi-rigid structures such as hexahydroindol-4-one (3*S*,6*S*)-diacid (S,S-9-O), which exhibits an unusually induced (between 50% and 70%) α-helix formation, as observed by circular dichroism (CD) when the ester-linked attached peptide chain is present. By contrast, the helix did not form when the peptide chain is coupled through an amide linker, since a 3_10_-type hydrogen-bonding pattern (*i, i+3*) is expected in the structure in this condition (Austin et al., 1997).

Alternative approaches involve template motifs designed to elongate and replicate spacing by incorporating groups of atoms containing carbonyls, thus resembling the arrangement found in other amino acid sequences. For instance, we can cite only one enantiomer of a complex bicyclic structure, with three carbonyl groups, which presents an increase in the peptides’ helicity anchored at the *N*-terminus (Mahon and Arora, 2012).

Other strategies are post-translational modifications, for example, *N*-methylation and *N*-alkylation (Petri et al., 2022), sulfonation, and the addition of phosphate groups, which can be carried out using known amino acid residues to gain chemical diversity, but also to improve activity or availability (King et al., 2021). The bioconjugation strategy is employed making use of natural amino acids with nucleophilic chains such as lysine and cysteine (Hoyt et al., 2019), but also non-canonical amino acids with bioorthogonal groups, such as azides, ketones, and alkynes, which allow subsequent chemical modification (Lang and Chin, 2014).

Stapling has been designed to establish a connection between side chains of natural or non-natural amino acids, forming at a certain space a cyclic structure that stabilizes the molecule, preserving a certain conformation and structure, while limiting its flexibility (Chu et al., 2015; Fairlie and Dantas de Araujo, 2016). This strategy has used modified amino acids containing side chains with terminal vinyl groups that allow, through Ru catalysts, a cyclic structure containing the new double bond, a bridge produced by olefin metathesis reactions. These cross-links can be added at positions *i, i+3*, and *i, i+4* to obtain one helical turn, but also between positions *i, i+7*, bridging two helical turns (Chu et al., 2015; Cromm et al., 2015). To favor helical structures using olefins side chains, bridges are installed at positions *i, i+3* (with six or eight carbon atoms connector); and another at position *i, i+7*, involving a cross-link with 11 carbon atoms. In order to achieve this, it is necessary that the building blocks have an *R* configuration at position *i* and an *S* configuration at position *i + n*. For the *i, i+4* bridge, the most used architecture involves an eight-carbon cross-link derived from two *S*-configured building blocks (Chu et al., 2015). The bioconjugation of cysteines into peptides has also been used for the optimization of them as well as proteins. The sulfhydryl groups from cysteines and an appropriate bifunctional linker allow the cross-link, carried out in solution with unprotected peptides, through two steps. The first step involves one cysteine reacting with the linker to form a linear mono thioether intermediate, followed by an intramolecular ring closure involving the second cysteine and the linker to give the stapling. The advantage of using cysteines for stapling, in relation to amino acids with vinyl groups as radicals, is their easy incorporation into heterologously produced peptide sequences (Fairlie and Dantas de Araujo, 2016). While this strategy is commonly used to establish peptide sequences as helices for enhancing protein-protein interactions (Timmerman et al., 2005; Verdine and Hilinski, 2012; Cromm et al., 2015), stapling insertion can also serve as initiators and stabilizers, creating capping effects, when positioned at the beginning and end of a given peptide sequence. Several studies describing the introduction of staple have found an improvement in some properties, such as providing high levels of helical, strong protection from proteolytic degradation (Shi et al., 2013), improves the bioavailability (Bird et al., 2010), robust cell-penetration and increase in target affinity (Verdine and Hilinski, 2012; Findeisen et al., 2017; Verhoork et al., 2019).

As well as in defensins and other cysteine-rich peptides, which have motifs such as α-helices and antiparallel β-sheets typically stabilized by disulfide bonds (Cociancich et al., 1993; Ehret-Sabatier et al., 1996; Zhu and Gao, 2013), it is possible to generate cyclic and synthetic peptidomimetics, with cysteines and form disulfide bonds to link the sequence through sites, either head-to-tail or head-to-center, favoring or not a specific structure. Cyclization generally helps stabilize the secondary structure and preserve a specific bioactive conformation due to confinement within a rigid structure. However, in certain cases such as some grafted peptides, this constraint may lead to a reduction in antimicrobial activity as it hinders their ability to interact and insert into pathogen membranes or intracellular targets (Rezende et al., 2021). Therefore, the design of this type of peptide can include some intrinsic variables found in the sequences of AMPs with disulfide bonds. The function of such bridges is to maintain different stable motifs within the tertiary structures, since it is believed that these conformations are important to perform a certain recognition function in specific receptors, playing a beneficial role for the organism that produces them (Hogg, 2009).

The exchange of some functional groups for bioisosteres in N- or C-capping regions in linear peptides also leads to improved molecules that can both improve physicochemical characteristics and enhance activity (Ding et al., 2023; Zhan et al., 2023). Furthermore, non-peptide fractions coupled to N-cap tripeptides are active against viral serine proteases, which result in excellent inhibitors of the aforementioned enzyme (Nitsche et al., 2012; 2013).

## 4 Modulation of α-helix structures by amino acid motif interactions into *N*- and *C*-terminus

According to data in the Protein Data Bank (PDB), α-helices constitute 57% of experimentally identified proteins, as reported on the RCSB PDB website (https://www.rcsb.org/). These helices are a predominant secondary structure commonly found in globular proteins (Wang et al., 2022), and play a pivotal role in molecular recognition (Bajpayee et al., 2023). In many proteins, the α-helix motif serves as a recognition domain by directly binding to other macromolecules (Guharoy and Chakrabarti, 2007). Using these helical structures as a basis for optimizing AMPs could serve as an additional feature, because this approach might potentially broaden its impact by influencing intracellular receptors, thereby improving its efficacy. This is particularly significant since numerous AMPs appear to operate through interaction with microbial membranes, and not through protein-like receptors.

The motivation to comprehend the process of α-helix formation stems from the aspiration to design and develop stable and uniquely folded α-helices, demonstrating novel biological functions and/or therapeutic applications (Acharyya et al., 2019). In a short peptide sequence, nucleation is a higher energy step and involves the organization of the initial three amino acids into a helical turn, facilitated by specific stabilizing interactions (Mahon and Arora, 2012).

Other non-covalent interactions such as metal-ligand, designed host-guest interactions, salt bridges, cation-π interactions, and π-π stacking are important to the α-helix stabilization, which can be achieved due to the presence of appropriately spaced residues in the peptide chain (Yin, 2012). The spatial distribution of amino acid residues along the folded peptide can form discrete portions, which have hydrophobic or hydrophilic properties (Kabelka and Vácha, 2021). In addition to the fact that helical structures are often established in solution for many peptides, a significant portion of them present the α-helical structure and are enhanced in contact with target membranes (Koehbach and Craik, 2019).

A study investigating the influence of salt bridges on helix formation found that the polyalanine peptide AEP ((Ala)_9_-Arg-(Ala)_3_-Glu-(Ala)_4_-Arg-(Ala)_2_) stabilizes its α-helix structure through a salt bridge between the side chains of residues Arg-10 and Glu-14 at *i* and *i+4* positions, respectively (Hong et al., 2011). However, the replacement of Glu-14 with Arg in AP ((Ala)_8_-Arg-(Ala)_4_-Arg-(Ala)_4_-Arg-(Ala)_2_) did not increase the stability of the α-helix, as it remained similar to the α-helix of the original AEP, but with some different contributions (Hong et al., 2011). The difference between the helices shows that in the α-helix length distribution AEP is exposed to more numerous but shorter length α-helix segments, which means that AEP has an increased concentration of α-helix-turn-α-helix conformations (Hong et al., 2011).

In addition, certain motifs are crucial for AMPs’ secondary structure formation, such as the nucleolin Thr-Pro-Ala-Lys-Lys motif, in the peptides TP1 (Ac-Gly-Ala-Thr-Pro-Ala-Lys-Lys-Ala-Ala-Gly-NH_2_) and TP2 (Ac-Gly-Ala-Thr-Pro-Ala-Lys-Lys-Ala-Ala-Ala-Thr-Pro-Ala-Lys-Lys-Ala-Ala-Gly-NH_2_). At high pH and in the presence of trifluoroethanol, both peptides adopt a helical structure, likely stabilized via N-capping, with the threonine and proline sequence initiating short helical segments in the motif. Analysis of the nuclear Overhauser effect (NOE) spectra indicates that the helix starts from Pro. However, Thr interacts with the side chain of the first Lys through two NOE interactions at (*i, i+2*): NH(Thr) to H-β (Lys), and NH(Thr) to NH(Lys). Consequently, the rest of the structure is stabilized by the uncharged side chain of Lys (Xu et al., 1995).

Recently, we reported the effect of the Asn-Lys-Pro motif as an N-cap in the peptide PaDBS1R7 (Pro-Met-Ala-Arg-Asn-Lys-Pro-Lys-Ile-Leu-Lys-Arg-Ile-Leu-Ala-Lys-Ile-Phe-Lys). It was observed from nuclear magnetic resonance (NMR) data that Asn-5 has a crucial role in stabilizing the α-helix, as a coil/N-cap/α-helix structural scaffold, through hydrogen bonds formed between the side chain of Asn and amino acid in the main chain (Lys6, Pro7, Ile9 and Leu10). The N-cap effect is mainly driven through Asn-5(NH in the side chain)/Lys-6(NH), (*i, i+1*), and Asn-5(NH in the side chain)/Leu-10(NH), (*i, i+5*). This peptide eradicated *Pseudomonas aeruginosa* biofilms as well as showing decreased bacterial counts by 100–1000-fold *in vivo* using a skin abscess mouse model (Cardoso et al., 2022).

Examination of both the ^3^ *J*NHαH coupling constant and the NOE experiments demonstrated that the X-Leu-Leu-Arg-Ala motif, originating from the *N*-terminal segment of the leucine zipper (LZ)-like domain of the HIV envelope gp41 glycoprotein—where X denotes a group or amino acid residue capable of forming in van der Waals interactions, hydrophobic interactions and/or hydrogen bond with an arginine—functions as the nucleating core for the helix in four synthetic decapeptides (Chang et al., 2000). Understanding the role of amino acid residues in the *N*-terminal region is important for the development of α-helical peptides. Asparagine is considered the best residue for the *N*-terminal region to stabilize the helix, while glycine is good, and glutamine is the worst residue for this position (Chakrabartty et al., 1993). In fact, researchers suggest using helicogenic residues to stabilize helical conformations, such as the introduction of Asp and Lys to form lactam bridges (*i, i+4*) (Barazza et al., 2005; Mimna et al., 2007).

In particular cases, stereochemical aspects also play a significant role in the ability to form α-helical peptides. For example, the replacement of *N*-terminal capping amino acid residues Lys-Leu-Thr of peptide QK (Ac-Lys-Leu-Thr-Trp-Gln-Glu-Leu-Tyr-Gln-Leu-Lys-Tyr-Lys-Gly-Ile-NH_2_), a vascular endothelial growth factor (VEGF) mimetic short helical peptide, with the corresponding *D* enantiomers (^*D*^Lys-^*D*^Leu-^*D*^Thr) negatively affected its ability to structure into an α-helix, which is fundamental to its biological activity (De Rosa et al., 2022).

Another important factor for helix formation is the size of the side chain of amino acid residues such as Arg, for example,. Tests over Ala-based peptides with *N*-terminal acetylation and *C*-terminal amidation were conducted to evaluate the effect of side chain length of Arg and Arg analogues ((*S*)-2-amino-6-guanidinohexanoic acid (Agh), (*S*)-2-amino-4-guanidinobutyric acid (Agb) and (*S*)-2-amino-3-guanidinopropionic acid (Agp)), as N-cap and C-cap. The results demonstrated that all four peptides were unfavorable for N-capping. The C-capping parameter followed the trend Agp < Agb < Arg < Agh, showing more favorable C-cap energetics with increasing side chain length (Cheng et al., 2012). By contrast, the propensity for helix formation showed a tendency Agp < Agb > Arg > Agh, highlighting the singularity and importance of the Arg side chain and analogs for helix formation, in the C-cap region (Cheng et al., 2012).

Various peptides were synthesized based on repetitions of the sequence (Arg-Leu-Leu-Arg)_n_, (n = 2–5), because this motif is considerate a α-helix former. Among these, the peptide (Arg-Leu-Leu-Arg)_5_ exhibits this characteristic and has minimum inhibitory concentrations (MICs) between 1 and 4 mM against microorganisms (gram-negative bacteria, gram-positive bacteria, and fungi); however, its effectiveness is reduced 32-fold in high salt conditions (100 or 200 mM NaCl). Posteriorly, the Ala-Pro-Lys-Ala-Met and Leu-Gln-Lys-Lys-Gly-Ile motifs were added to the repetitions, in the *N*- and *C*-terminals, respectively. These last motifs have amphipathic characteristics, a positive net charge, and show interactions between the residues that allow nucleation peptides. The *N*-terminal motif (Ala-Pro-Lys-Ala-Met) presents a hydrophobic interaction between the side chains of Ala and Met (*i, i+4*). Meanwhile, the *C*-terminal Leu-Gln-Lys-Lys-Gly-Ile motif forms two H-bonds: one between the Leu amide group and the Ile carbonyl group (*i, i+5*), and another between the amide of the Gln side chain and the carbonyl group of Gly (*i, i+3*). Interestingly, with the addition of two capping motifs into the two peptide repetitions (Ala-Pro-Lys-Ala-Met-Arg-Leu-Leu-Arg-Arg-Leu-Leu-Arg-Leu-Gln-Lys-Lys-Gly), the integrity of the MIC values (0.5 and 2 μg.mL^−1^ for gram negative, gram-positive bacteria, and yeast) was observed at NaCl concentrations equal to 200 mM, and its helicity was not compromised. These motifs have the property of stabilizing the helix, maintaining the structural integrity and antimicrobial activity of the peptide at high salt concentrations (Park et al., 2004).

The synthetic peptide (Leu-Leu-Lys-Lys)_2_-NH_2_ was modified by insertion of a Cys residue at the *C*-terminus and in both terminal regions. Those modifications resulted in an enhanced α-helix structure and significantly increased antibacterial activity against *B. subtilis* (125 mg.L^−1^ for both), compared to the parental peptide (500 mg.L^−1^). Additionally, the presence of thiol groups in the modified peptide enhances its antimicrobial potency against gram-positive bacteria and yeast. Moreover, it broadened its activity spectrum to include gram-negative bacteria, exhibiting potency levels of >500 mg.L^−1^ against *E. coli* and 63 mg.L^−1^ against *P. aeruginosa* for (Leu-Leu-Lys-Lys)_2_Cys, and 225 mg.L^−1^ against *E. coli* and 125 mg.L^−1^ against *P. aeruginosa* for Cys-(Leu-Leu-Lys-Lys)_2_-Cys. Notably, the latter peptide exhibited enhanced pore formation (Wiradharma et al., 2011). Furthermore, this peptide effectively eradicated clinically isolated carbapenem-resistant *Acinetobacter baumannii* in mouse models of peritonitis and pneumonia infections (Huang et al., 2012), and it also exhibited activity *in vitro* against susceptible and multidrug-resistant clinical isolates of *M. tuberculosis* (Khara et al., 2014).

The *C*-terminal region is also important for stabilizing the α-helix and, consequently, maintaining activity, particularly when a polar side chain may be taking place. In a study by Kallenbach and Gong, peptides (based on the parental sequence Tyr-Met-Ser-Glu-Asp-Glu-Leu-Lys-Ala-Ala-Glu-Ala-Ala-Phe-Lys-Arg-His-Gly-Pro-Thr) were stabilized with an *N*-terminal region containing a classical Ser-X-X-Glu capping box (Ser-Glu-Asp-Glu motif), while several different capping motifs were tested near the *C*-terminal region to evaluate stabilization capacity (Kallenbach and Gong, 1999). The capping box, Ser-X-X-Glu, was identified by Harper and Rose, as a helix initiator component in the *N*-terminus of proteins (Harper and Rose, 1993). This motif involves two hydrogen-bonding interactions between the side chain and the main chain: (*i, i+3*). The first occurs between the Ser side chain and the amide of Glu, and the second occurs between the Glu side chain and the amide of Ser (Harper and Rose, 1993). Among substitutions in the three last amino acids at the *C*-terminus, an exceptionally strong interaction occurs when an Asn-18 residue is present in the Asn-Pro-Thr motif, interacting through its amide group in the side chain with the carbonyl group of the Phe-14 three residues away, forming a 3_10_-helix. This interaction (*i, i+4*) produces a greater propensity for helicity in this case. By contrast, the side chain of Gly-18 in the Gly-Val-Pro motif, also in the *C*-terminus, forms a double main chain–main chain H-bond with Phe-14 (Gly-18/Phe-14; (*i, i+4*)) and Lys15 (Gly-18/Lys-15; (*i, i+3*)). Here, the Pro-20 enhances the structure through nonpolar interaction of its side chain by interacting with the aromatic ring of Phe-14, collectively stabilizing the *N*-terminal capping box (Kallenbach and Gong, 1999).

The *C*-terminal Lys can structure a peptide with three amino acids as a helix. This was demonstrated by Zabuga and Rizzo (2015), when they detected a helix in the sequence Ac-Phe-Ala-LysH^+^. In this case, the Lys side chain forms three hydrogen bonds, where each hydrogen of the ammonia group bonds to the carbonyls of the main chain, similar to what occurs in polyalanine helices (Zabuga and Rizzo, 2015).

Evidence suggests that edge-face, offset-stacked or face-to-face stacked aromatic interaction also plays a crucial role in the α-helical monomeric structure. Edge-face and offset-stacked geometric interactions are preferred between two phenylalanine residues located *i* and *i+4*, respectively, whereas fully stacked geometry is observed when phenylalanine - pentafluorophenylalanine are in a similar position. In both cases, the interactions can stabilize helix formation in a prototype polyalanine peptide, but the interactions are stronger when the residues, in the same positions, are more towards the *C*-terminal region. The prevalence of helical structures and, consequently the diverse activities observed in AMPs, is significantly influenced by the interaction between rotamer populations of the aromatic chains. The interaction, particularly at the *C*-terminal region, enhances the motif’s effectiveness in promoting α-helices through the C-capping effect (Butterfield et al., 2002; Koehbach and Craik, 2019).

Although capping motifs can give us answers about the nucleation of the formation of α-helical structures, there are other factors that influence the stability of these conformations so that the role of complexes formed by interactions with metal ions can also generate another part of this answer.

## 5 Modulation of activity of AMPs by formation complexes with divalent ions

The role of metal ions cannot be underestimated, since studies with different peptides, proteins, and non-peptide molecules demonstrate their activity in intermolecular interactions within cellular physiological processes. Additionally, they play a role in enhancing the activity of molecules in the immune system. In the last decade, several studies of complexes formed by motifs of AMPs with divalent metal ions, such as Zn^2+^ and Cu^2+^ (Łoboda et al., 2018), have demonstrated the role of these ions in boosting the antimicrobial activity of these substances. This subfamily within the AMPs is called metallo-AMPs and has attracted attention because it has allowed the importance of this complex in the immune system to be demonstrated. Such potentiation may be related to the fact that the binding of an AMP to the metal removes the availability of the microbe to the ion, causing it to suffer due to the deficit of the metal. Alternatively, this complexation could improve the structure or charge of the peptide (Łoboda et al., 2018).

The classification of the relationships between metal cations and AMPs has been described as falling into three classes, explaining the modes of action. However, it is essential to note that this classification is not unique, and there may be other forms. The system was primarily based on Zn^2+^, as the relationship between Zn^2+^-AMPs has provided sufficient information to establish their synergistic relationship. Hence, class I is distinguished by the modulation of AMP activity through metal ion binding. In this class, cations can act as a cofactor that either enhances or inhibits the antimicrobial activity of AMPs. In class II, AMPs may regulate cation availability within the host. This implies that AMPs can either enable the availability of ions if the host restricts them from the microbe or increase the concentration of the cation within the pathogen, leading to toxicity. In class III, cations may indirectly influence the activity of an AMP, and the existence of an ion-AMP complex is not a requirement. In this scenario, the cation enhances the activity of an AMP by inhibiting the microbe’s responses (Donaghy et al., 2023).

Among the mechanisms mentioned earlier, we can highlight class I, which is the most commonly observed in certain AMPs and Zn^2+^ ions. Dermcidin-derived peptides belong to a family resulting from the proteolysis processes of dermcidin (DCD), a protein expressed by sweat glands. These peptides play a role in the skin’s immune defense system, and within them, DCD-1 and DCD-1L are the major ones. Both peptides have demonstrated efficacy against both gram negative and gram-positive bacteria (*E. coli*, *Enterococcus faecalis*, and *S. aureus*), as well as yeasts like *C. albicans*, in buffer solutions with conditions of pH, ionic strength, and sodium, potassium, magnesium and chlorine ions, which simulate human sweat conditions (Schittek et al., 2001). Structurally, these two peptides present a wide range of residues with polar groups (-OH) and donors of protons (-COOH), the latter being acidic groups that confer a total charge of −2 at neutral pH. Both peptides adopt an undefined random conformation in aqueous solution; however, DCD-1L adopts an α-helix structure in the presence of surfactants that mimic the cell membrane of gram-negative bacteria (Steffen et al., 2006; Paulmann et al., 2012). The mechanism of action of dermcidin-derived peptides is attributed to the formation of oligomerization (composed of dimers and trimers), a structure believed to be important in antibacterial activity. This oligomerization structuring has been observed in an *in vitro* experiment involving DCD-1L in human sweat, and it seems that Zn^2+^ ions play a crucial role in stabilizing the oligomeric structure and in the boosting activity. Activity reduction becomes apparent upon the addition of a chelating agent, such as EDTA (Paulmann et al., 2012).

**FIGURE 1:**
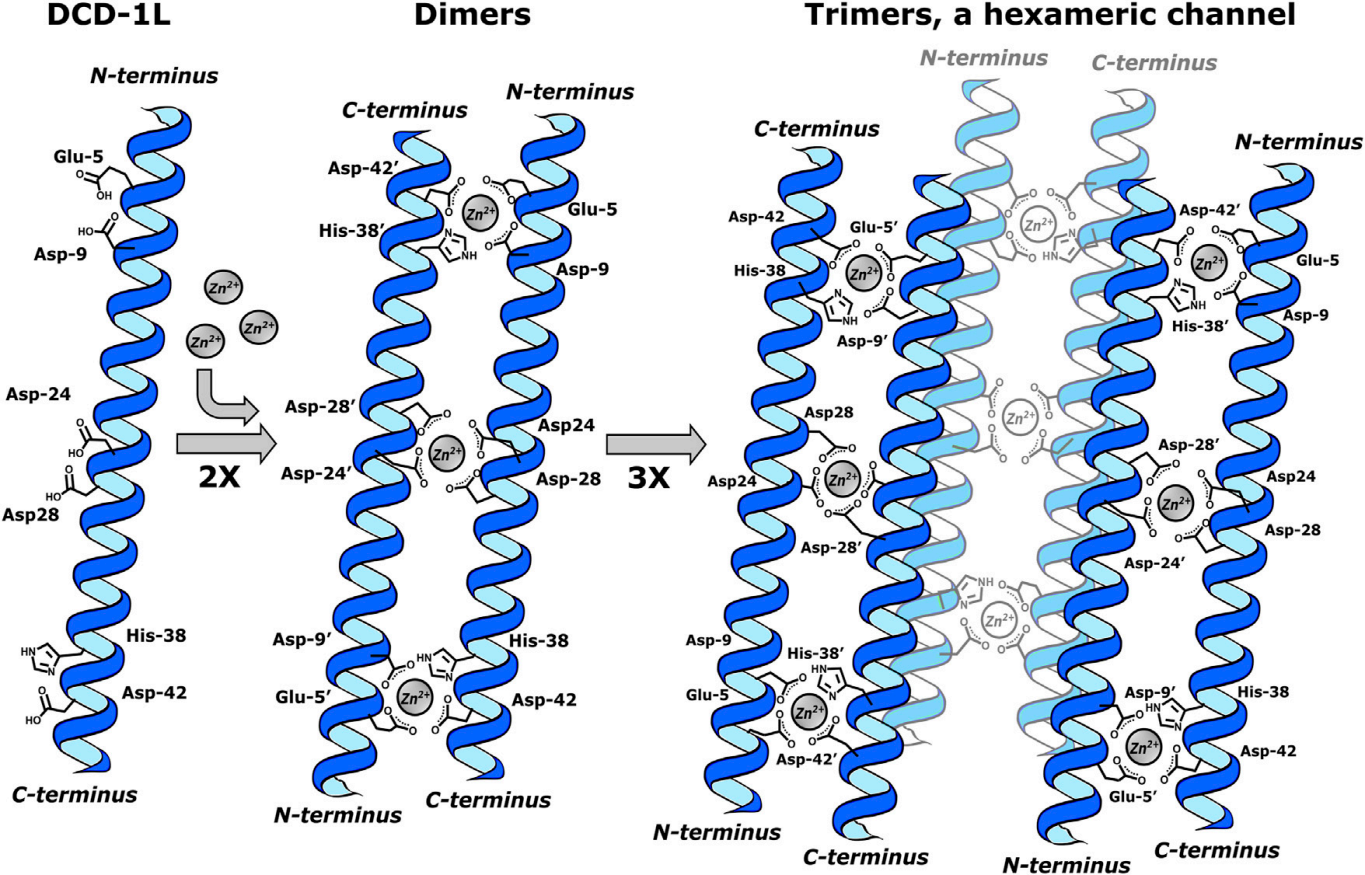
DCD-1L forming a trimer of Zn^2+^ bridge dimers, a hexameric channel. Dimers are formed by paired peptide helices (2X) oriented in head-to-tail directions and coordinated to two Zn^2+^ ions through Glu-5, Asp-9 (*i, i+4* and *i, i+9* motifs at *N*-terminus) and His-38′ and Asp-42’ (*i, i+38′* together with *i, i+42′* in *C*-terminal). The formation of trimers occurs through the Asp-24/Asp-28 and Asp-24’/Asp-28′ in the center of the helix, which coordinate with one Zn^2+^ion. This last interaction stabilizes each dimer in the trimer. This figure was created using Inkscape, version 1.3.0.

Dermcidin-derived peptides, with DCD-1L (Ser-Ser-Leu-Leu-Glu-Lys-Gly-Leu-Asp-Gly-Ala-Lys-Lys-Ala-Val-Gly-Gly-Leu-Gly-Lys-Leu-Gly-Lys-Asp-Ala-Val-Glu-Asp-Leu-Glu-Ser-Val-Gly-Lys-Gly-Ala-Val-His-Asp-Val-Lys-Asp-Val-Leu-Asp-Ser-Val-Leu) as a significant constituent, exhibit a mechanism of action attributed to class I. In this process, Zn^2+^ ions play a crucial role in structuring oligomers, forming “ion channels” or pores in bacterial membranes (Paulmann et al., 2012). The structuring was carried out in a solution of 1-palmitoyl-2-oleoyl-sn-glycero-3-phosphoethanolamine (POPE)/1-palmitoyl-2-oleoyl-sn-glycero-3-phospho (1′-rac-glycerol) (POPG) in a molar ratio 3:1. The elucidation by X-ray diffraction (XRD), solid-state nuclear magnetic resonance (ssNMR), and molecular dynamics (MD) shows that DCD-1L forms a trimer of Zn^2+^ bridge dimers. Each dimer is formed by paired peptide helices oriented in head-to-tail directions and coordinated to Zn^2+^ ions as the central atom via Glu-5, Asp-9 (*i, i+4* and *i, i+9* motifs at *N*-terminus), and His-38′ and Asp-42’ (*i, i+38′* together with *i, i+42′* in *C*-terminal). For the formation of trimers, stabilization of the dimers occurs through the pairs Asp-24/Asp-28 and Asp-24’/Asp-28′ in the center of the helix, which coordinate with Zn^2+^. In total, six polypeptide chains form a hexameric channel that creates a pore resembling a barrel inside the membrane. The Zn^2+^:DCD-1L complex was observed to form in a 1:1 ratio, where all Zn^2+^ ions neutralize the charge of the hexamer (Figure 1) (Song et al., 2013).

Likewise, certain motifs in AMPs that exhibit binding to transition metals have been reported. Generally, such interactions are attributed to residues with neutral, basic, or acidic polar side chains in AMPs, which contain heteroatoms capable of coordinating with these metals. As an example, we can cite the His-Gly-Phe-Ser-His motif found at the *C*-terminus, between amino acids 17–21 of the peptide clavanin A (Clav-A: Val-Phe-Gln-Phe-Leu-Gly-Lys-Ile-Ile-His-His-Val-Gly-Asn-Phe-Val-His-Gly-Phe-Ser-His-Val-Phe-NH_2_) (Duay et al., 2019). Clav-A is a representative within the group of peptides known as clavanins, isolated from the tunicate *Styela clava*. Cationic, amphipathic and rich in His, it is an α-helix-forming peptide, and has a broad spectrum of antimicrobial action against bacteria and fungi. It distinguishes itself by its wide-ranging efficacy against methicillin-resistant *Staphylococcus aureus* (MRSA) at pH 5.5. At physiological pH (pH 7.4), it shows less effectiveness, but it also exhibits activity under high salt conditions. These data suggest that Clav-A has different modes of action under different pH and salt conditions (Lee et al., 1997). In the presence of Zn^2+^ ions, the activity of Clav-A is increased 16-fold, and it seems that this increase is derived from the stabilization of this ion in the His-Gly-Phe-Ser-His motif, where His-17 and His-21 are located (*i*, *i+4*) and each one is responsible for coordination. Molecular modeling on the Zn^2+^-Clav-A system in membrane environments demonstrates that the mechanism of action may be attributed to the coordination, providing strong electrostatic interactions between the complex (His-Gly-Phe-Ser-His motif at the *C*-terminus of Clav-A) and the lipid layer, leading to membrane dissociation (Duay et al., 2019).

At physiological pH, Clav-A can independently engage with the membrane of gram-negative bacteria, subsequently leading to membrane distortion and changes in cellular functionality, such as the inhibition of division in *E. coli* (Juliano et al., 2020). In contrast, at acidic pH, Clav-A seems to exhibit two mechanisms of action. The first involves binding to ionophores, deactivating their function, and facilitating the transfer of ions between the cytoplasm and the extracellular environment. This enables Zn^2+^ ions to enter the cell, causing Clav-A to act similarly to indolicin, which inhibits DNA synthesis. Additionally, there may be a translocation of the peptide, Zn^2+^, and/or Zn^2+^-Clav-A complex across the membrane (Juliano et al., 2017). In the latter case, the Zn^2+^-Clav-A complex may influence cytoplasmic DNA through a mechanism proposed by Juliano et al. (2020). The mechanism, investigated through quantum mechanics/molecular mechanics (QM/MM) calculations, suggested that Zn^2+^, in the Zn^2+^-Clav-A complex, acts as a Lewis acid, activating the P-O bonds in DNA. A hydroxyl group from water then nucleophilically attacks the electrophytic phosphorus, cleaving the P-O bond activated by Zn^2+^. The breakage of the phosphoester bond hydrolyzes bacterial DNA (Juliano et al., 2020).

**FIGURE 2:**
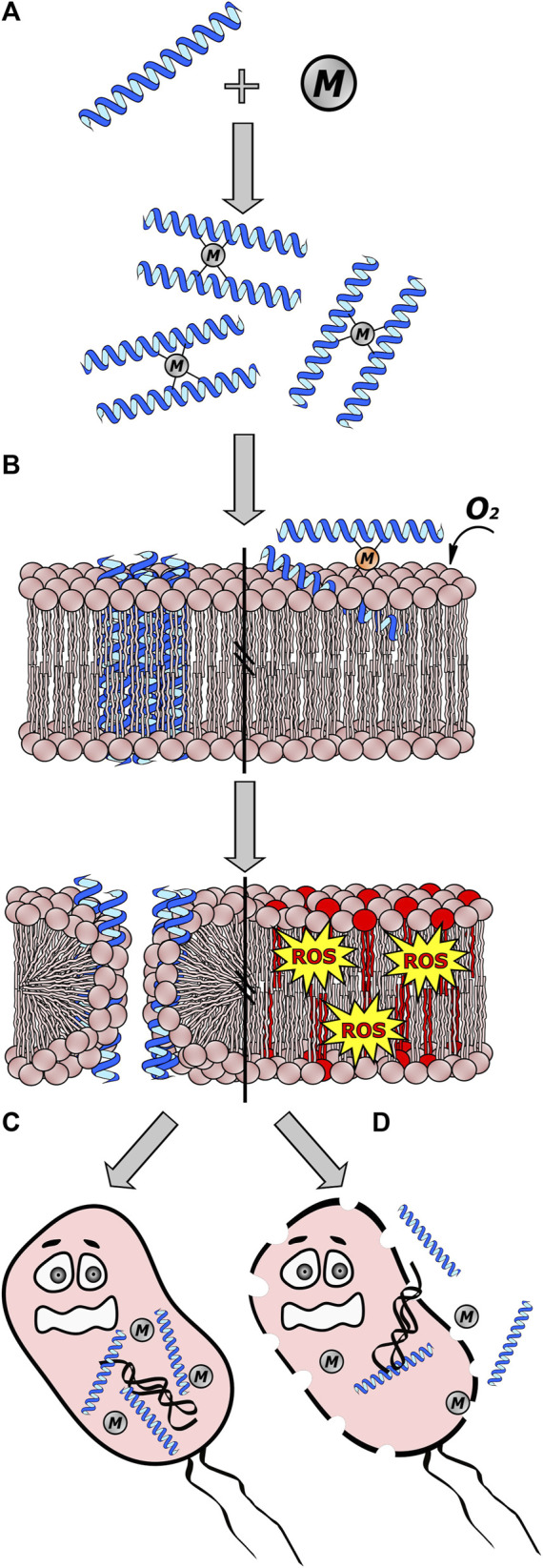
Interaction between antimicrobial peptides (AMPs) and metal ions (M). In class I interactions, certain cations can either enhance or inhibit the antimicrobial activity of AMPs by acting as a cofactor. **(A)** Some metallic ions bind to AMPs through specific amino acids, resulting in the formation of dimers. **(B)** The M-AMP complex can then interact with the cell membrane, forming pores or evolving into more complex structures by joining dimers to create channels. Additionally, certain complexes like Cu^2+^ ions and AMPs with the ATCUN motif can lead to the production of reactive oxygen species (ROS) through oxygen-dependent lipid peroxidation of phospholipids, ultimately causing cell membrane disintegration. **(C)** These channels formed allow the passage of AMPs and/or metal ions into the cell, where they can interact with intracellular targets. **(D)** If AMPs fail to interact with intracellular targets, pore and channel formation can destabilize the cell membrane, resulting in cell death and the leakage of cellular material. This figure was created using Inkscape, version 1.3.0.

A complex of divalent copper (Cu^2+^) and nickel (Ni^2+^) ions in amino-terminal motifs, called (ATCUN), has been found in specific AMPs. These motifs are thought to be essential in boosting the oxidative action mechanism (Alexander et al., 2017). One such examples is ixosine, a peptide isolated from the tick *Ixodes sinensis*, which exhibits a similar effect on the lipid membranes of bacteria. The mechanism also appears to be class I, according to the classification by Donaghy et al. (2023), through a strategy employed by the tick in using Cu^2+^ ions as part of the activation of its immune defense against bacterial infections. In the case of ixosine (Gly-Leu-His-Lys-Val-Met-Arg-Glu-Val-Leu-Gly-Tyr-Glu-Arg-Asn-Ser-Tyr-Lys-Lys-Phe-Phe-Leu-Arg), the first three amino acids (Gly-Leu-His) are the motif responsible and that can coordinate to Cu^2+^ and Ni^2+^. Similar motifs like NH_2_-AA_1_-AA_2_-His, are found in proteins such as albumins and protamines, which coordinate these two metals through the terminal amino group, the -NH- group of AA_2_, and the imidazole ring of histidine (Melino et al., 1999; Libardo et al., 2016). In ticks, it seems that the Cu^2+^-ixosine complex plays a role in mediating molecular oxygen-dependent lipid peroxidation of phospholipids to produce reactive oxygen species (ROS). The authors theorize that these ROS leads to the intramolecular formation of aldehyde-type carbonyl compounds, derivatives capable of reacting with amino groups of other AMPs, such as ixosine B, for example, to form Schiff bases. This allows the binding of AMPs in the membrane, forming helices as they interact closely with the membrane to create pores (Figure 2) (Libardo et al., 2016).

Using an *in silico* approach, our group previously redesigned the AMPs, CM15 (Lys-Trp-Lys-Leu-Phe-Lys-Lys-Ile-Gly-Ala-Val-Leu-Lys-Val-Leu-NH_2_), and citropin1.1 (Gly-Leu-Phe-Asp-Val-Ile-Lys-Lys-Val-Ala-Ser-Val-Ile-Gly-Gly-Leu-NH_2_), by the addition of ATCUN motifs (Gly-Gly-His or Val-Ile-His) at their *N*-terminus. It is noteworthy that both motifs, when inserted into CM15, were shown to enhance activity against carbapenem-resistant *Klebsiella pneumoniae* (KpC+ 1,825,971) by 4-fold for Gly-Gly-His-CM15 and 8-fold for His-Ile-Val-CM15. By contrast, modification of both ATCUN motifs in citropin1.1 resulted in a 3-fold decrease in antimicrobial activity when tested against both gram negative and gram-positive bacteria. CD spectra for CM15 peptides containing both ATCUN motifs showed an increase in helicity in the absence of Cu^2+^ (Agbale et al., 2019).

Histatins are small peptides released by the parotid and sub-mandibular salivary gland in both humans and primates. These peptides are generally cationic and rich in histidine, playing a key role in antimicrobial activity against bacteria and fungi, wound healing and the buccal immune system (Tay et al., 2009; Puri and Edgerton, 2014). Among the most potent is Histatin 5 (Hst-5: Asp-Ser-His-Ala-Lys-Arg-His-His-Gly-Tyr-Lys-Arg-Lys-Phe-His-Glu-Lys-His-His-Ser-His-Arg-Gly-Tyr), resulting from the proteolytic degradation of histatin 1 and 3. Hst-5 is active against *Cryptococcus neoformans, Aspergillus fumigatu, Candida albicans* and other yeasts (Helmerhorst et al., 1999). It also exhibits activity against multiple-drug-resistant pathogens (ESKAPE: *Enterococcus faecium*, *S. aureus*, *K. pneumoniae*, *A. baumannii*, *Pseudomona*s *aeruginosa*, and *Enterobacter* species), causing hospital or nosocomial infections (Du et al., 2017). Hst-5 presents part of an ACTUN motif (Asp-Ser-His) in its *N*-terminal as well as the His-X-X-X-His-His motif in its *N*-terminal and the His-Glu-X-X-His motif in its *C*-terminal for Zn^2+^ coordination. Apparently, the union of Zn^2+^ and Cu^2+^ in the respective motifs confers stabilization on the α-helix structure (Cragnell et al., 2019; McCaslin et al., 2019), and data from several studies on the metal-Hst-5 complex suggest significant consequences for increased activity. In the work by Melino et al. (1999), it was observed that the Zn^2+^-Hst-5 complex exhibits a catalytic effect on the fusion of negatively charged lipid vesicles via a mechanism of action that involves electrostatic interactions mediated by the formation of dimers (Melino et al., 1999). Additionally, Zn^2+^ ions have shown to increase the bactericidal activity of histidine-rich peptides against *E. faecalis* (Rydengård et al., 2006).

The formation of ROS has been proven through several experiments where the complex is formed between Cu^2+^ and peptides containing ATCUN, such as those of Hst-5. For example, by mass spectrometry, oxidized derivatives of a peptide analogue of Hst-5 (P1: Asp-Ser-His-Ala-Lys-Arg-Ala-His-Gly-Tyr) were detected after the addition of ascorbic acid to a 1:1 stoichiometry solution of Cu^2+^:P1 complex. After 5 min of exposure to the reductant, it was possible to identify the presence of adduct ions with an increase of 16 and 32 Da in the mass of P1 (Cabras et al., 2007). Additionally, the same copper complex, using Hst-5 or Hst-8 (Lys-Phe-His-Glu-Lys-His-His-Ser-His-Arg-Gly-Tyr), revealed an increase in ROS production, including hydrogen peroxide, at physiological concentrations of ascorbic acid in *vitro* experiments. Here, Houghton and Nicholas (2009) observed the production of hydrogen peroxide using the Amplex Red assay, by incubating at 20°C for 60 min the peptide stock solutions (Hst-5 or Hst-8) containing copper chloride and buffered ascorbate. The Amplex Red assay is based on the oxidation of 10-acetyl-3,7-dihydroxypenoxazine, which is catalyzed by horseradish peroxidase (HRP) in the presence of H_2_O_2_ to produce a red fluorescent oxidation product, which is monitored at 570 nm (Houghton and Nicholas, 2009). This finding further supports the effects of ROS production on bacteria killing (Libardo et al., 2016), and control of the metal-peptide complex in candidiasis infections. In *C. albicans*, mitochondria produce superoxide dismutase (Sod5) dependent on Cu/Zn/Mn, which is induced under conditions of oxidative stress. The enzymatic activity of yeast can be a target due to the transmetalation of such ions mediated by Hst-5 (Martchenko et al., 2004). The complex between Zn^2+^-Hst-5 is similar to that formed by DCD-1L and ClavA, with 2:2 stoichiometry, where each Zn^2+^ ion coordinates with four histidine residues, two of which are in a polypeptide chain of Hst-5. Some studies suggest that Hst-5 activity in *Candida* species is dependent on Zn^2+^ concentration since the low ratio (0.5:1 or less) of the metal in the complex has been shown to have improved antifungal activity in comparison with the peptide alone. This improvement can be attributed to cellular reorganization induced by Zn^2+^, decreasing the ability to adhere to the cell wall of the host (Norris et al., 2020). Nevertheless, at higher ratios (1:1 or higher), Zn^2+^ ions seem to impede the antifungal activity of Hst-5 against *C. albicans* (Campbell et al., 2022). According to Stewart et al. (2023), both higher and lower proportions of Zn^2+^ in the Zn^2+^-Hst-5 complex do not exert an effect on improving or suppressing the effect on multiple species of *Streptococcus*, which normally colonize the oral cavity. Additionally, Hst-5 does not cause Zn^2+^ starvation in this genus, as it does not compete for Zn^2+^ binding with the Zn^2+^ uptake protein AdcAI (Stewart et al., 2023). The above study, along with others, suggests that Hst-5 may play a role in the development of certain species of oral cavity microorganisms, exerting selective antimicrobial activity and maintaining microbial communities regulated through Zn^2+^ ion concentrations (Norris et al., 2021; Campbell et al., 2022; Stewart et al., 2023). With all this evidence, Hst-5 cannot be classified as belonging to class II; instead, it appears to be able to act in class I or III.

Calcitermin (Val-Ala-Ile-Ala-Leu-Lys-Ala-Ala-His-Tyr-His-Thr-His-Lys-Glu) corresponds to the last 15 residues at the *C*-terminus of calgranulin C, a member of the S100 family of antibacterial proteins produced by neutrophils, monocytes, and keratinocytes (Gottsch et al., 1999; D’Accolti et al., 2023). This AMP shows activity against several pathogens (*E. coli, P. aeruginosa, S. aureus, S. epidermidis, E. faecalis, C. albicans,* and *L. monocytogenes*), depending on the conditions (Cole et al., 2001). For example, at neutral pH (7.4), it does not show activity, but at acidic pH (5.4), it is active against *E. coli, P. aeruginosa*, *E. faecalis,* and *C. albicans* (Cole et al., 2001; Bellotti et al., 2019). In the presence of Zn^2+^ ions, its activity increases against *E. coli* and is effective against *L. monocytogenes* (Cole et al., 2001). Coordination of two metals, Zn^2+^ and Cu^2+^, occurs effectively at acidic pH, using His-9, His-11, His-13, and different amino acid residues for each metal. In the case of the Zn^2+^ complex, the carboxylate group of the side chain of Glu-15 participates, but with Cu^2+^, the other ligand is the *N*-terminal group of Val-1. This coordination improves the activity of the peptide against *C. albicans*, as the MIC decreases to 1 μg.mL^−1^ for both complexes. Meanwhile, the complex with Cu^2+^ maintains activity against *S. aureus* in the same way as non-complexed calcitermin. CD experiments for the Cu^2+^-Calcitermin complex, and Cu^2+^ with three other analogs (substitution of each His with an Ala in the parental Calcitermin), reveal that the metal in all complexes helps to adopt a helical-like structure in the presence of membrane-mimicking sodium dodecyl sulfate (SDS) (Bellotti et al., 2019).

Another peptide that relies on the presence of divalent ions is Bacitracin. Isolated as a mixture of cyclic dodecapeptides from *Bacillus* species, it contains both *D*- and *L*-amino acids and is synthesized by nonribosomal peptide synthases (Economou et al., 2013). Among these peptides, bacitracin A is the main constituent, exhibiting the highest effectiveness against bacteria. It is selective for gram-positive bacteria and shows limited activity against gram-negative bacteria. It has been classified as class I according to Donaghy et al. (2023) due to its binding with Zn^2+^ ions, forming a complex that enhances the activity of the cyclopeptide (Donaghy et al., 2023). The Zn^2+^-Bacitracin complex binds to a lipid intermediate (undecaprenyl pyrophosphate), an important transporter in the biosynthesis of the bacterial cell wall. The binding of the complex to the intermediate interrupts the flow of peptidoglycan precursors, leading to the inhibition of cell wall formation and, consequently, negatively affecting bacterial development (Economou et al., 2013). The stoichiometry of the complex formed between Zn^2+^-bacitracin-undecaprenyl pyrophosphate has been reported as 1:1:1. These data were suggested by crystallization experiments performed on the Zn^2+^-bacitracin-geranyl pyrophosphate complex. In this complex, Zn^2+^ coordinates mainly with the *N*-terminal region of this AMP, and geranyl pyrophosphate is also involved. Specifically, bacitracin engages both geranyl pyrophosphate and Zn^2+^ ions in the coordination process. In this coordination, bacitracin employs the terminal amino group, the nitrogen on the thiazoline ring, and the carboxylate of Glu-4. By contrast, the lipid participates use the oxygens of the pyrophosphate group, which, together with a water molecule, assume an octahedral geometry. In this case, unlike the previous examples, the coordination of Zn^2+^ by the His present in the bacitracin macrocycle is not observed (Economou et al., 2013).

Kappacin (106–169) is the non-glycosylated and phosphorylated form of the caseinomacropeptide (CMP) derived from bovine milk k-casein (the *C*-terminal fragment between residues 106 and 169). This AMP exhibits activity against the oral opportunistic pathogen *Streptococcus mutans* and demonstrates a membranolytic effect observed in experiments with artificial liposomes, exhibiting pH-dependent behavior. At acidic pH (6.5), it has the ability to permeabilize liposomes; however, at neutral pH (7.2), it exhibits little effect on them, as evidenced by the lack of antibacterial activity. However, the addition of a metal: Kappacin (106–169) ratio (2:1) for Ca^2+^ or (1:1) for Zn^2+^ results in improved antibacterial activity at neutral pH. Divalent metal cation binding assays and Scatchard analyses indicated that Kappacin (106–169) has two binding sites for the metal. Proton nuclear magnetic resonance (^1^H-NMR) experiments on the Kappacin (138–158) fragment, conducted in the presence of Ca^2+^ ions and 30% tetrafluoroethylene (TFE), suggest that the peptide adopts a specific conformation in this environment (Dashper et al., 2005). The sequence Kappacin (138–158) (Ala-Val-Glu-Ser-Thr-Val-Ala-Thr-Leu-Glu-Asn-Ser-Pro-Glu-Val-Ile-Glu-Ser-Pro-Pro-Glu) is rich in Glu spaced (*i, i+4*) at the *C*-terminus, which may explain the coordination with divalent ions, thus potentially modulating the peptide structuration.

A new field, known as metallo-AMPs, has emerged from the interaction between divalent metal ions and specific residues within AMPs, warranting further exploration. As mentioned in the observed cases, this interaction acts by increasing effectiveness against microorganisms and arises from the coordination of divalent ions along the structure of AMPs, enhancing the secondary structure and performing various functions in the face of microbial targets. This interaction is expected, as both metallic ions and AMPs are considered essential components of the immune system in various organisms.

## 6 Concluding remarks and prospects

Capping motifs play an important role in modulating the antimicrobial activity, selectivity, and protease resistance in AMPs. Beyond direct antimicrobial properties, presumably by promoting the structuring of peptide sequences in the α-helix, such as specific sequences and divalent cations, capping motifs may influence other desirable properties, such as *in vivo* stability and biocompatibility. Cationic amino acids, such as Lys and Arg, have demonstrated efficacy as capping motifs because charge at the *N*- or *C*-terminus helps enhance interactions with negatively charged microbial membranes while reducing cytotoxicity against mammalian cells. Some internal interactions of the side chains of these residues, as well as unique and unusually structured motifs, within the peptide chain, contribute to the nucleation and structuring of α-helices, which are crucial for biological activity.

However, further research is needed to fully elucidate the structure-activity relationships between capping motifs and the AMP’s mechanism of action. The results from studies of molecular dynamics simulations and biophysical techniques, in which it was explored how specific capping motifs alter AMP secondary structure, oligomerization, membrane perturbation abilities, and the capping motifs’ performance against a wider range of clinically relevant and multidrug-resistant pathogens, can be used in new approaches based on artificial intelligence and deep machine learning, to generate new structural data, as well as new active peptides.

Through continued progress in understanding capping motif-mediated effects, it may be possible to customize AMPs for specific infection types or drug delivery applications, helping to address the pressing need for novel anti-infectives, particularly in the face of escalating antimicrobial resistance crises worldwide. Capping motifs offer a promising design element for to developing next-generation AMP therapeutics with improved efficacy, safety, and pharmacological profiles.

## References

[B1] Acharyya A. Ge Y. Wu H. Degrado W. F. Voelz V. A. Gai F. (2019). Exposing the nucleation site in α-helix folding: a joint experimental and simulation study. J. Phys. Chem. B 123, 1797–1807. 10.1021/acs.jpcb.8b12220 30694671 PMC6497059

[B2] Agbale C. M. Sarfo J. K. Galyuon I. K. Juliano S. A. Silva G. G. O. Buccini D. F. (2019). Antimicrobial and antibiofilm activities of helical antimicrobial peptide sequences incorporating metal-binding motifs. Biochemistry 58, 3802–3812. 10.1021/acs.biochem.9b00440 31448597

[B3] Alexander J. L. Yu Z. Cowan J. A. (2017). Amino terminal copper and nickel binding motif derivatives of ovispirin-3 display increased antimicrobial activity via lipid oxidation. J. Med. Chem. 60, 10047–10055. 10.1021/acs.jmedchem.7b01117 29172482

[B4] Aurora R. Rose G. D. (1998). Helix capping. Protein Sci. 7, 21–38. 10.1002/pro.5560070103 9514257 PMC2143812

[B5] Austin R. E. Maplestone R. A. Sefler A. M. Liu K. Hruzewicz W. N. Liu C. W. (1997). A template for stabilization of a peptide α-helix: synthesis and evaluation of conformational effects by circular dichroism and NMR. J. Am. Chem. Soc. 119, 6461–6472. 10.1021/ja964231a

[B6] Babii O. Afonin S. Schober T. Komarov I. V. Ulrich A. S. (2017). Flexibility vs rigidity of amphipathic peptide conjugates when interacting with lipid bilayers. Biochim. Biophys. Acta Biomembr. 1859, 2505–2515. 10.1016/j.bbamem.2017.09.021 28958778 PMC5667891

[B7] Bajpayee N. Vijayakanth T. Rencus-Lazar S. Dasgupta S. Desai A. V. Jain R. (2023). Exploring helical peptides and foldamers for the design of metal helix frameworks: current trends and future perspectives. Angew. Chem. - Int. Ed. 62, e202214583. 10.1002/anie.202214583 36434750

[B8] Barazza A. Wittelsberger A. Fiori N. Schievano E. Mammi S. Toniolo C. (2005). Bioactive N-terminal undecapeptides derived from parathyroid hormone: the role of *α* -helicity. J. Peptide Res. 65, 23–35. 10.1111/j.1399-3011.2005.00207.x 15686531

[B9] Behrouz S. Kühl N. Klein C. D. (2023). N-sulfonyl peptide-hybrids as a new class of dengue virus protease inhibitors. Eur. J. Med. Chem. 251, 115227. 10.1016/j.ejmech.2023.115227 36893626

[B10] Bellotti D. Toniolo M. Dudek D. Mikołajczyk A. Guerrini R. Matera-Witkiewicz A. (2019). Bioinorganic chemistry of calcitermin-the picklock of its antimicrobial activity. Dalton Trans. 48, 13740–13752. 10.1039/c9dt02869b 31475275

[B11] Bird G. H. Madani N. Perry A. F. Princiotto A. M. Supko J. G. He X. (2010). Hydrocarbon double-stapling remedies the proteolytic instability of a lengthy peptide therapeutic. Proc. Natl. Acad. Sci. 107, 14093–14098. 10.1073/pnas.1002713107 20660316 PMC2922607

[B12] Butterfield S. M. Patel P. R. Waters M. L. (2002). Contribution of aromatic interactions to α-helix stability. J. Am. Chem. Soc. 124, 9751–9755. 10.1021/ja026668q 12175233

[B13] Cabras T. Patamia M. Melino S. Inzitari R. Messana I. Castagnola M. (2007). Pro-oxidant activity of histatin 5 related Cu(II)-model peptide probed by mass spectrometry. Biochem. Biophys. Res. Commun. 358, 277–284. 10.1016/j.bbrc.2007.04.121 17482573

[B14] Campbell J. X. Gao S. Anand K. S. Franz K. J. (2022). Zinc binding inhibits cellular uptake and antifungal activity of histatin-5 in Candida albicans. ACS Infect. Dis. 8, 1920–1934. 10.1021/acsinfecdis.2c00289 35997625 PMC9671271

[B15] Cardoso M. H. Chan L. Y. Cândido E. S. Buccini D. F. Rezende S. B. Torres M. D. T. (2022). An N-capping asparagine-lysine-proline (NKP) motif contributes to a hybrid flexible/stable multifunctional peptide scaffold. Chem. Sci. 13, 9410–9424. 10.1039/d1sc06998e 36093022 PMC9383710

[B16] Chakrabartty A. Doig A. J. Baldwin R. L. (1993). Helix capping propensities in peptides parallel those in proteins. Proc. Natl. Acad. Sci. 90, 11332–11336. 10.1073/pnas.90.23.11332 8248248 PMC47976

[B17] Chang D.-K. Cheng S.-F. Yang S.-H. (2000). A helix initiation motif, XLLRA, is stabilized by hydrogen bond, hydrophobic and van der Waals interactions. Biochimica Biophysica Acta (BBA) - Protein Struct. Mol. Enzym. 1478, 39–50. 10.1016/S0167-4838(99)00286-1 10719173

[B18] Cheng R. P. Weng Y.-J. Wang W.-R. Koyack M. J. Suzuki Y. Wu C.-H. (2012). Helix formation and capping energetics of arginine analogs with varying side chain length. Amino Acids 43, 195–206. 10.1007/s00726-011-1064-2 21922267

[B19] Chu H. L. Yu H. Y. Yip B. S. Chih Y. H. Liang C. W. Cheng H. T. (2013). Boosting salt resistance of short antimicrobial peptides. Antimicrob. Agents Chemother. 57, 4050–4052. 10.1128/AAC.00252-13 23716061 PMC3719776

[B20] Chu Q. Moellering R. E. Hilinski G. J. Kim Y. W. Grossmann T. N. Yeh J. T. H. (2015). Towards understanding cell penetration by stapled peptides. Medchemcomm 6, 111–119. 10.1039/c4md00131a

[B21] Cociancich S. Goyffon M. Bontems F. Bulet P. Bouet F. Menez A. (1993). Purification and characterization of a scorpion defensin, a 4kDa antibacterial peptide presenting structural similarities with insect defensins and scorpion toxins. Biochem. Biophys. Res. Commun. 194, 17–22. 10.1006/bbrc.1993.1778 8333834

[B22] Cole A. M. Kim Y. H. Tahk S. Hong T. Weis P. Waring A. J. (2001). Calcitermin, a novel antimicrobial peptide isolated from human airway secretions. FEBS Lett. 504, 5–10. 10.1016/S0014-5793(01)02731-4 11522286

[B23] Cragnell C. Staby L. Lenton S. Kragelund B. B. Skepö M. (2019). Dynamical oligomerisation of histidine rich intrinsically disordered proteins is regulated through zinc-histidine interactions. Biomolecules 9, 168. 10.3390/biom9050168 31052346 PMC6571702

[B24] Cromm P. M. Spiegel J. Grossmann T. N. (2015). Hydrocarbon stapled peptides as modulators of biological function. ACS Chem. Biol. 10, 1362–1375. 10.1021/cb501020r 25798993

[B25] D’Accolti M. Bellotti D. Dzień E. Leonetti C. Leveraro S. Albanese V. (2023). Impact of C- and N-terminal protection on the stability, metal chelation and antimicrobial properties of calcitermin. Sci. Rep. 13, 18228. 10.1038/s41598-023-45437-0 37880318 PMC10600247

[B26] Dashper S. G. O’Brien-Simpson N. M. Cross K. J. Paolini R. A. Hoffmann B. Catmull D. V. (2005). Divalent metal cations increase the activity of the antimicrobial peptide kappacin. Antimicrob. Agents Chemother. 49, 2322–2328. 10.1128/AAC.49.6.2322-2328.2005 15917528 PMC1140507

[B27] Dennison S. R. Phoenix D. A. (2011). Influence of C-terminal amidation on the efficacy of modelin-5. Biochemistry 50, 1514–1523. 10.1021/bi101687t 21241054

[B28] de Oliveira K. B. S. Lopes Leite M. Albuquerque Cunha V. Brito da Cunha N. Luiz Franco O. (2023). Challenges and advances in antimicrobial peptide development. Drug Discov. Today 28, 103629. 10.1016/j.drudis.2023.103629 37230283

[B29] De Rosa L. Diana D. Capasso D. Stefania R. Di Stasi R. Fattorusso R. (2022). Switching the N-capping region from all-L to all-D amino acids in a VEGF mimetic helical peptide. Molecules 27, 6982. 10.3390/molecules27206982 36296575 PMC9607104

[B30] Ding D. Xu S. da Silva-Júnior E. F. Liu X. Zhan P. (2023). Medicinal chemistry insights into antiviral peptidomimetics. Drug Discov. Today 28, 103468. 10.1016/j.drudis.2022.103468 36528280

[B31] Donaghy C. Javellana J. G. Hong Y. J. Djoko K. Angeles-Boza A. M. (2023). The synergy between zinc and antimicrobial peptides: an insight into unique bioinorganic interactions. Molecules 28, 2156. 10.3390/molecules28052156 36903402 PMC10004757

[B32] dos Santos Cabrera M. P. Rangel M. Ruggiero Neto J. Konno K. (2019). Chemical and biological characteristics of antimicrobial α-helical peptides found in solitary wasp venoms and their interactions with model membranes. Toxins (Basel) 11, 559. 10.3390/toxins11100559 31554187 PMC6832458

[B33] Du H. Puri S. McCall A. Norris H. L. Russo T. Edgerton M. (2017). Human salivary protein histatin 5 has potent bactericidal activity against ESKAPE pathogens. Front. Cell Infect. Microbiol. 7, 41. 10.3389/fcimb.2017.00041 28261570 PMC5309243

[B34] Duay S. S. Sharma G. Prabhakar R. Angeles-Boza A. M. May E. R. (2019). Molecular dynamics investigation into the effect of zinc(II) on the structure and membrane interactions of the antimicrobial peptide clavanin A. J. Phys. Chem. B 123, 3163–3176. 10.1021/acs.jpcb.8b11496 30908921 PMC6545919

[B35] Economou N. J. Cocklin S. Loll P. J. (2013). High-resolution crystal structure reveals molecular details of target recognition by bacitracin. Proc. Natl. Acad. Sci. U. S. A. 110, 14207–14212. 10.1073/pnas.1308268110 23940351 PMC3761639

[B36] Ehret-Sabatier L. Loew D. Goyffon M. Fehlbaum P. Hoffmann J. A. van Dorsselaer A. (1996). Characterization of novel cysteine-rich antimicrobial peptides from scorpion blood. J. Biol. Chem. 271, 29537–29544. 10.1074/jbc.271.47.29537 8939880

[B37] Fairlie D. P. Dantas de Araujo A. (2016). Stapling peptides using cysteine crosslinking. Biopolymers 106, 843–852. 10.1002/bip.22877 27178225

[B38] Fernandes F. C. Cardoso M. H. Gil-Ley A. Luchi L. V. da Silva M. G. L. Macedo M. L. R. (2023). Geometric deep learning as a potential tool for antimicrobial peptide prediction. Front. Bioinforma. 3, 1216362. 10.3389/fbinf.2023.1216362 PMC10374423 37521317

[B39] Findeisen F. Campiglio M. Jo H. Abderemane-Ali F. Rumpf C. H. Pope L. (2017). Stapled voltage-gated calcium channel (CaV) α-interaction domain (AID) peptides act as selective protein-protein interaction inhibitors of CaV function. ACS Chem. Neurosci. 8, 1313–1326. 10.1021/acschemneuro.6b00454 28278376 PMC5481814

[B40] Fu Q. Cao D. Sun J. Liu X. Li H. Shu C. (2023). Prediction and bioactivity of small-molecule antimicrobial peptides from Protaetia brevitarsis Lewis larvae. Front. Microbiol. 14, 1124672. 10.3389/fmicb.2023.1124672 37007486 PMC10060639

[B41] Gottsch J. D. Eisinger S. W. Liu S. H. Scott A. L. (1999). Calgranulin C has filariacidal and filariastatic activity. Infect. Immun. 67, 6631–6636. 10.1128/IAI.67.12.6631-6636.1999 10569784 PMC97076

[B42] Guharoy M. Chakrabarti P. (2007). Secondary structure based analysis and classification of biological interfaces: identification of binding motifs in protein-protein interactions. Bioinformatics 23, 1909–1918. 10.1093/bioinformatics/btm274 17510165

[B43] Hack V. Reuter C. Opitz R. Schmieder P. Beyermann M. Neudörfl J.-M. (2013). Efficient α-helix induction in a linear peptide chain by N -capping with a bridged-tricyclic diproline analogue. Angew. Chem. Int. Ed. 52, 9539–9543. 10.1002/anie.201302014 23873809

[B44] Harper E. T. Rose G. D. (1993). Helix stop signals in proteins and peptides: the capping box. Biochemistry 32, 7605–7609. 10.1021/bi00081a001 8347570

[B45] Helmerhorst E. J. Reijnders I. M. van ’t Hof W. Simoons-Smit I. Veerman E. C. I. Amerongen A. V. N. (1999). Amphotericin B- and fluconazole-resistant *Candida* spp., *Aspergillus fumigatus*, and other newly emerging pathogenic fungi are susceptible to basic antifungal peptides. Antimicrob. Agents Chemother. 43, 702–704. 10.1128/AAC.43.3.702 10049295 PMC89188

[B46] Hogg P. J. (2009). Contribution of allosteric disulfide bonds to regulation of hemostasis. J. Thrombosis Haemostasis 7, 13–16. 10.1111/j.1538-7836.2009.03364.x 19630758

[B47] Hong Z. Ahmed Z. Asher S. A. (2011). Circular dichroism and ultraviolet resonance Raman indicate little arg-glu side chain α-helix peptide stabilization. J. Phys. Chem. B 115, 4234–4243. 10.1021/jp112238q 21425805 PMC3074482

[B48] Houghton E. A. Nicholas K. M. (2009). *In vitro* reactive oxygen species production by histatins and copper(I,II). J. Biol. Inorg. Chem. 14, 243–251. 10.1007/s00775-008-0444-x 18975018

[B49] Hoyt E. A. Cal P. M. S. D. Oliveira B. L. Bernardes G. J. L. (2019). Contemporary approaches to site-selective protein modification. Nat. Rev. Chem. 3, 147–171. 10.1038/s41570-019-0079-1

[B50] Huang Y. Wiradharma N. Xu K. Ji Z. Bi S. Li L. (2012). Cationic amphiphilic alpha-helical peptides for the treatment of carbapenem-resistant Acinetobacter baumannii infection. Biomaterials 33, 8841–8847. 10.1016/j.biomaterials.2012.08.026 22925814

[B51] Jiang Y. Chen Y. Song Z. Tan Z. Cheng J. (2021). Recent advances in design of antimicrobial peptides and polypeptides toward clinical translation. Adv. Drug Deliv. Rev. 170, 261–280. 10.1016/j.addr.2020.12.016 33400958

[B52] Jiang Y. Wang R. Feng J. Jin J. Liang S. Li Z. (2023). Explainable deep hypergraph learning modeling the peptide secondary structure prediction. Adv. Sci. 10, e2206151. 10.1002/advs.202206151 PMC10104664 36794291

[B53] Juliano S. A. Pierce S. Demayo J. A. Balunas M. J. Angeles-Boza A. M. (2017). Exploration of the innate immune system of Styela clava: Zn2+ binding enhances the antimicrobial activity of the tunicate peptide clavanin A. Biochemistry 56, 1403–1414. 10.1021/acs.biochem.6b01046 28226206

[B54] Juliano S. A. Serafim L. F. Duay S. S. Heredia Chavez M. Sharma G. Rooney M. (2020). A potent host defense peptide triggers DNA damage and is active against multidrug-resistant gram-negative pathogens. ACS Infect. Dis. 6, 1250–1263. 10.1021/acsinfecdis.0c00051 32251582 PMC7772950

[B55] Kabelka I. Vácha R. (2021). Advances in molecular understanding of α-helical membrane-active peptides. Acc. Chem. Res. 54, 2196–2204. 10.1021/acs.accounts.1c00047 33844916

[B56] Kallenbach N. R. Gong Y. (1999). C-Terminal capping motifs in model helical peptides. Bioorg Med. Chem. 7, 143–151. 10.1016/S0968-0896(98)00231-4 10199664

[B57] Kanbayashi N. Kataoka Y. Okamura T. A. Onitsuka K. (2022). Stability enhancement of a π-stacked helical structure using substituents of an amino acid side chain: helix Formation via a nucleation-elongation mechanism. J. Am. Chem. Soc. 144, 6080–6090. 10.1021/jacs.2c01337 35325538

[B58] Kemp D. S. Curran T. P. Davis W. M. Boyd J. G. Muendel C. (1991). Studies of N-terminal templates for.alpha.-helix formation. Synthesis and conformational analysis of (2S,5S,8S,11S)-1-acetyl-1,4-diaza-3-keto-5-carboxy-10-thiatricyclo[2.8.1.04,8]tridecane (Ac-Hel1-OH). J. Org. Chem. 56, 6672–6682. 10.1021/jo00023a037

[B59] Kemp D. S. Rothman J. H. (1995). Efficient helix nucleation by a macrocyclic triproline-derived template. Tetrahedron Lett. 36, 4023–4026. 10.1016/0040-4039(95)00707-J

[B60] Khara J. S. Wang Y. Ke X. Y. Liu S. Newton S. M. Langford P. R. (2014). Anti-mycobacterial activities of synthetic cationic α-helical peptides and their synergism with rifampicin. Biomaterials 35, 2032–2038. 10.1016/j.biomaterials.2013.11.035 24314557

[B61] Kier B. L. Shu I. Eidenschink L. A. Andersen N. H. (2010). Stabilizing capping motif for β-hairpins and sheets. Proc. Natl. Acad. Sci. 107, 10466–10471. 10.1073/pnas.0913534107 20484672 PMC2890817

[B62] King T. A. Mandrup Kandemir J. Walsh S. J. Spring D. R. (2021). Photocatalytic methods for amino acid modification. Chem. Soc. Rev. 50, 39–57. 10.1039/d0cs00344a 33174541

[B63] Koehbach J. Craik D. J. (2019). The vast structural diversity of antimicrobial peptides. Trends Pharmacol. Sci. 40, 517–528. 10.1016/j.tips.2019.04.012 31230616

[B64] Lang K. Chin J. W. (2014). Cellular incorporation of unnatural amino acids and bioorthogonal Labeling of Proteins. Chem. Rev. 114, 4764–4806. 10.1021/cr400355w 24655057

[B65] Lee I. H. Cho Y. Lehrer R. I. (1997). Effects of pH and salinity on the antimicrobial properties of clavanins. Infect. Immun. 65, 2898–2903. 10.1128/iai.65.7.2898-2903.1997 9199465 PMC175407

[B66] Lei J. Sun L. Huang S. Zhu C. Li P. He J. (2019). The antimicrobial peptides and their potential clinical applications. Am. J. Transl. Res. 11, 3919–3931. 31396309 PMC6684887

[B67] Li X. Zuo S. Wang B. Zhang K. Wang Y. (2022). Antimicrobial mechanisms and clinical application prospects of antimicrobial peptides. Molecules 27, 2675. 10.3390/molecules27092675 35566025 PMC9104849

[B68] Libardo M. D. J. Gorbatyuk V. Y. Angeles-Boza A. M. (2016). Central role of the copper-binding motif in the complex mechanism of action of ixosin: enhancing oxidative damage and promoting synergy with ixosin B. ACS Infect. Dis. 2, 71–81. 10.1021/acsinfecdis.5b00140 27622949

[B69] Łoboda D. Kozłowski H. Rowińska-Zyrek M. (2018). Antimicrobial peptide-metal ion interactions-a potential way of activity enhancement. New J. Chem. 42, 7560–7568. 10.1039/c7nj04709f

[B70] Magana M. Pushpanathan M. Santos A. L. Leanse L. Fernandez M. Ioannidis A. (2020). The value of antimicrobial peptides in the age of resistance. Lancet Infect. Dis. 20, e216–e230. 10.1016/S1473-3099(20)30327-3 32653070

[B71] Mahon A. B. Arora P. S. (2012). End-capped α-helices as modulators of protein function. Drug Discov. Today Technol. 9, e57–e62. 10.1016/j.ddtec.2011.07.008 22712023 PMC3375709

[B72] Martchenko M. Alarco A. M. Harcus D. Whiteway M. (2004). Superoxide dismutases in Candida albicans: transcriptional regulation and functional characterization of the hyphal-induced SOD5 gene. Mol. Biol. Cell 15, 456–467. 10.1091/mbc.E03-03-0179 14617819 PMC329211

[B73] McCaslin T. G. Pagba C. V. Yohannan J. Barry B. A. (2019). Specific metallo-protein interactions and antimicrobial activity in Histatin-5, an intrinsically disordered salivary peptide. Sci. Rep. 9, 17303. 10.1038/s41598-019-52676-7 31754129 PMC6872563

[B74] Melino S. Rufini S. Sette M. Morero R. Grottesi A. Paci M. (1999). Zn2+ ions selectively induce antimicrobial salivary peptide histatin- 5 to fuse negatively charged vesicles. Identification and characterization of a zinc-binding motif present in the functional domain. Biochemistry 38, 9626–9633. 10.1021/bi990212c 10423240

[B75] Mimna R. Tuchscherer G. Mutter M. (2007). Toward the design of highly efficient, readily accessible peptide N-caps for the induction of helical conformations. Int. J. Pept. Res. Ther. 13, 237–244. 10.1007/s10989-006-9073-9

[B76] Mondal A. Lenz S. MacCallum J. L. Perez A. (2023). Hybrid computational methods combining experimental information with molecular dynamics. Curr. Opin. Struct. Biol. 81, 102609. 10.1016/j.sbi.2023.102609 37224642

[B77] Mura M. Wang J. Zhou Y. Pinna M. Zvelindovsky A. V. Dennison S. R. (2016). The effect of amidation on the behaviour of antimicrobial peptides. Eur. Biophysics J. 45, 195–207. 10.1007/s00249-015-1094-x PMC4796345 26745958

[B78] Mwangi J. Kamau P. Thuku R. Lai R. (2023). Design methods for antimicrobial peptides with improved performance. Zool. Res. 0, 0. 10.24272/j.issn.2095-8137.2023.246 PMC10802102 37914524

[B79] Nitsche C. Behnam M. A. M. Steuer C. Klein C. D. (2012). Retro peptide-hybrids as selective inhibitors of the Dengue virus NS2B-NS3 protease. Antivir. Res. 94, 72–79. 10.1016/j.antiviral.2012.02.008 22391061

[B80] Nitsche C. Schreier V. N. Behnam M. A. M. Kumar A. Bartenschlager R. Klein C. D. (2013). Thiazolidinone-peptide hybrids as dengue virus protease inhibitors with antiviral activity in cell culture. J. Med. Chem. 56, 8389–8403. 10.1021/jm400828u 24083834

[B81] Norris H. L. Kumar R. Edgerton M. (2021). A novel role for histatin 5 in combination with zinc to promote commensalism in c. Albicans survivor cells. Pathogens 10, 1609. 10.3390/pathogens10121609 34959564 PMC8703888

[B82] Norris H. L. Kumar R. Ong C. Y. Xu D. Edgerton M. (2020). Zinc binding by histatin 5 promotes fungicidal membrane disruption in c. Albicans and c. glabrata. J. Fungi 6, 124–216. 10.3390/jof6030124 PMC7559477 32751915

[B83] Ong Z. Y. Wiradharma N. Yang Y. Y. (2014). Strategies employed in the design and optimization of synthetic antimicrobial peptide amphiphiles with enhanced therapeutic potentials. Adv. Drug Deliv. Rev. 78, 28–45. 10.1016/j.addr.2014.10.013 25453271

[B84] Otvos L. Bokonyi K. Varga I. Ertl H. C. J. Hoffmann R. Bulet P. (2000). Insect peptides with improved protease‐resistance protect mice against bacterial infection. Protein Sci. 9, 742–749. 10.1110/ps.9.4.742 10794416 PMC2144618

[B85] Park I. Y. Cho J. H. Kim K. S. Kim Y.-B. Kim M. S. Kim S. C. (2004). Helix stability confers salt resistance upon helical antimicrobial peptides. J. Biol. Chem. 279, 13896–13901. 10.1074/jbc.M311418200 14718539

[B86] Pasupuleti M. Schmidtchen A. Chalupka A. Ringstad L. Malmsten M. (2009). End-tagging of ultra-short antimicrobial peptides by W/F stretches to facilitate bacterial killing. PLoS One 4, e5285. 10.1371/journal.pone.0005285 19381271 PMC2667214

[B87] Paul P. K. C. Sukumar M. Bardi R. Piazzesi A. M. Valle G. Toniolo C. (1986). Stereochemically constrained peptides. Theoretical and experimental studies on the conformations of peptides containing 1-aminocyclohexanecarboxylic acid. J. Am. Chem. Soc. 108, 6363–6370. 10.1021/ja00280a038

[B88] Paulmann M. Arnold T. Linke D. Özdirekcan S. Kopp A. Gutsmann T. (2012). Structure-activity analysis of the dermcidin-derived peptide DCD-1L, an anionic antimicrobial peptide present in human sweat. J. Biol. Chem. 287, 8434–8443. 10.1074/jbc.M111.332270 22262861 PMC3318687

[B89] Petri G. L. Di Martino S. De Rosa M. (2022). Peptidomimetics: an overview of recent medicinal chemistry efforts toward the discovery of novel small molecule inhibitors. J. Med. Chem. 65, 7438–7475. 10.1021/acs.jmedchem.2c00123 35604326

[B90] Puri S. Edgerton M. (2014). How does it kill?: understanding the candidacidal mechanism of salivary histatin 5. Eukaryot. Cell 13, 958–964. 10.1128/EC.00095-14 24951439 PMC4135785

[B91] Rezende S. B. Oshiro K. G. N. Júnior N. G. O. Franco O. L. Cardoso M. H. (2021). Advances on chemically modified antimicrobial peptides for generating peptide antibiotics. Chem. Commun. 57, 11578–11590. 10.1039/D1CC03793E 34652348

[B92] Rydengård V. Andersson Nordahl E. Schmidtchen A. (2006). Zinc potentiates the antibacterial effects of histidine-rich peptides against *Enterococcus faecalis* . FEBS J. 273, 2399–2406. 10.1111/j.1742-4658.2006.05246.x 16704414

[B93] Santini A. Barone V. Bavoso A. Benedetti E. Di Blasio B. Fraternali F. (1988). Structural versatility of peptides from Cα,α-dialkylated glycines: a conformational energy calculation and X-ray diffraction study of homopeptides from 1-aminocyclopentane-1-carboxylic acid. Int. J. Biol. Macromol. 10, 292–299. 10.1016/0141-8130(88)90007-4

[B94] Schittek B. Hipfel R. Sauer B. Bauer J. Kalbacher H. Stevanovic S. (2001). Dermcidin: a novel human antibiotic peptide secreted by sweat glands. Nat. Immunol. 2, 1133–1137. 10.1038/ni732 11694882

[B95] Schmidtchen A. Pasupuleti M. Mörgelin M. Davoudi M. Alenfall J. Chalupka A. (2009). Boosting antimicrobial peptides by hydrophobic oligopeptide end tags. J. Biol. Chem. 284, 17584–17594. 10.1074/jbc.M109.011650 19398550 PMC2719397

[B96] Serrano L. Fersht A. R. (1989). Capping and α-helix stability. Nature 342, 296–299. 10.1038/342296a0 2812029

[B97] Shi X. Wales T. E. Elkin C. Kawahata N. Engen J. R. Annis D. A. (2013). Hydrogen exchange-mass spectrometry measures stapled peptide conformational dynamics and predicts pharmacokinetic properties. Anal. Chem. 85, 11185–11188. 10.1021/ac403173p 24215480 PMC3883098

[B98] Song C. Weichbrodt C. Salnikov E. S. Dynowski M. Forsberg B. O. Bechinger B. (2013). Crystal structure and functional mechanism of a human antimicrobial membrane channel. Proc. Natl. Acad. Sci. U. S. A. 110, 4586–4591. 10.1073/pnas.1214739110 23426625 PMC3607029

[B99] Spicer V. Lao Y. W. Shamshurin D. Ezzati P. Wilkins J. A. Krokhin O. V. (2014). N-capping motifs promote interaction of amphipathic helical peptides with hydrophobic surfaces and drastically alter hydrophobicity values of individual amino acids. Anal. Chem. 86, 11498–11502. 10.1021/ac503352h 25372782

[B100] Steffen H. Rieg S. Wiedemann I. Kalbacher H. Deeg M. Sahl H. G. (2006). Naturally processed dermcidin-derived peptides do not permeabilize bacterial membranes and kill microorganisms irrespective of their charge. Antimicrob. Agents Chemother. 50, 2608–2620. 10.1128/AAC.00181-06 16870749 PMC1538671

[B101] Stewart L. J. Hong Y. J. Holmes I. R. Firth S. J. Ahmed Y. Quinn J. (2023). Salivary antimicrobial peptide histatin-5 does not display Zn(II)-Dependent or -independent activity against streptococci. ACS Infect. Dis. 9, 631–642. 10.1021/acsinfecdis.2c00578 36826226 PMC10012264

[B102] Svenson J. Stensen W. Brandsdal B. O. Haug B. E. Monrad J. Svendsen J. S. (2008). Antimicrobial peptides with stability toward tryptic degradation. Biochemistry 47, 3777–3788. 10.1021/bi7019904 18307313

[B103] Tay W. M. Hanafy A. I. Angerhofer A. Ming L. J. (2009). A plausible role of salivary copper in antimicrobial activity of histatin-5-Metal binding and oxidative activity of its copper complex. Bioorg Med. Chem. Lett. 19, 6709–6712. 10.1016/j.bmcl.2009.09.119 19846304

[B104] Timmerman P. Beld J. Puijk W. C. Meloen R. H. (2005). Rapid and quantitative cyclization of multiple peptide loops onto synthetic scaffolds for structural mimicry of protein surfaces. ChemBioChem 6, 821–824. 10.1002/cbic.200400374 15812852

[B105] Tossi A. Sandri L. Giangaspero A. (2000). Amphipathic, α-helical antimicrobial peptides. Biopolymers 55, 4–30. 10.1002/1097-0282(2000)55:1<4::AID-BIP30>3.0.CO;2-M 10931439

[B106] Valle G. Crisma M. Toniolo C. Sen N. Sukumar M. Balaram P. (1988). Crystallographic characterization of the conformation of the 1-aminocyclohexane-1-carboxylic acid residue in simple derivatives and peptides. J. Chem. Soc. Perkin Trans. 2, 393. 10.1039/p29880000393

[B107] Verdine G. L. Hilinski G. J. (2012). Stapled peptides for intracellular drug targets. Methods in enzymology 503, 3–33. 10.1016/B978-0-12-396962-0.00001-X 22230563

[B108] Verhoork S. J. M. Jennings C. E. Rozatian N. Reeks J. Meng J. Corlett E. K. (2019). Tuning the binding affinity and selectivity of perfluoroaryl-stapled peptides by cysteine-editing. Chem. - A Eur. J. 25, 177–182. 10.1002/chem.201804163 30255959

[B109] Wang L. Wang N. Zhang W. Cheng X. Yan Z. Shao G. (2022). Therapeutic peptides: current applications and future directions. Signal Transduct. Target Ther. 7, 48–27. 10.1038/s41392-022-00904-4 35165272 PMC8844085

[B110] Wiradharma N. Khan M. Yong L. K. Hauser C. A. E. Seow S. V. Zhang S. (2011). The effect of thiol functional group incorporation into cationic helical peptides on antimicrobial activities and spectra. Biomaterials 32, 9100–9108. 10.1016/j.biomaterials.2011.08.020 21906803

[B111] Xu X. Cooper L. G. DiMario P. J. Nelson J. W. (1995). Helix formation in model peptides based on nucleolin TPAKK motifs. Biopolymers 35, 93–102. 10.1002/bip.360350110 7696559

[B112] Yin H. (2012). Constrained peptides as miniature protein structures. ISRN Biochem. 2012, 1–15. 10.5402/2012/692190 PMC4392992 25969758

[B113] Yue L. Song L. Zhu S. Fu X. Li X. He C. (2024). Machine learning assisted rational design of antimicrobial peptides based on human endogenous proteins and their applications for cosmetic preservative system optimization. Sci. Rep. 14, 947. 10.1038/s41598-023-50832-8 38200054 PMC10781772

[B114] Zabuga A. V. Rizzo T. R. (2015). Capping motif for peptide helix formation. J. Phys. Chem. Lett. 6, 1504–1508. 10.1021/acs.jpclett.5b00407 26263303

[B115] Zhan W. Li D. Saha P. Wang R. Zhang H. Ajay A. K. (2023). Discovery of highly selective inhibitors of the human constitutive proteasome β5c chymotryptic subunit. J. Med. Chem. 66, 1172–1185. 10.1021/acs.jmedchem.2c00733 36608337 PMC10157300

[B116] Zhu S. Gao B. (2013). Evolutionary origin of β-defensins. Dev. Comp. Immunol. 39, 79–84. 10.1016/j.dci.2012.02.011 22369779

